# Inducible manipulation of motor–cargo interaction using engineered kinesin motors

**DOI:** 10.1242/jcs.258776

**Published:** 2021-08-03

**Authors:** Jessica J. A. Hummel, Casper C. Hoogenraad

**Affiliations:** 1 Cell Biology, Neurobiology and Biophysics, Department of Biology, Faculty of Science, Utrecht University, Utrecht 3584 CH, The Netherlands; 2 Department of Neuroscience, Genentech, Inc., South San Francisco, CA 94080, USA

**Keywords:** Organelle, Kinesin, Neurons, Transport, Chemical induction

## Abstract

Molecular motors drive long-range intracellular transport of various vesicles and other cargoes within a cell. Identifying which kinesin motors interact with which type of transport vesicles has been challenging, especially in complex neuronal cells. Here, we present a highly adaptable toolbox of engineered kinesin motors to control and interrogate the selectivity and regulation of cargo transport with acute chemical induction. Selectivity of cargo–motor interaction can be addressed by systematic screening of a library of kinesin tails and neuronal cargoes. Additionally, our toolbox can be used to study kinesin–cargo regulatory mechanisms, and we found that cargo trafficking by KIF16B is regulated by its PX domain. Furthermore, our toolbox enables acute manipulation of polarized trafficking in living neurons by steering transport into axons or dendrites. Engineering kinesin motors provides a powerful tool to map the specificity of interactions between kinesin and cargoes, manipulate polarized transport and investigate cargo–motor interaction modes.

## Abstract (precis)

**Summary:** Development of an engineered kinesin motor platform that can be used to study kinesin–cargo interaction and manipulate polarized cargo trafficking, to provide an adaptable tool to study kinesin motor proteins in neurons.

## INTRODUCTION

Intracellular transport of cargo, including protein complexes, organelles and mRNA, is of great importance for cell development and survival. This becomes especially clear in highly polarized cells, such as neurons, which rely on long-distance transport for their survival. In neurons, newly synthesized cargoes need to be transported from their site of synthesis, the cell body, to their destination in the axon and dendrites. Simultaneously, retrograde transport is required to remove aging proteins and organelles, and to enable retrograde signaling by cargoes such as neurotrophic factors (Maday et al., 2014). Defects in this long-distance transport have been linked to many neurodegenerative diseases, including Alzheimer's disease and Parkinson's disease (Millecamps and Julien, 2013; Encalada and Goldstein, 2014).

Neurons are highly polarized cells typically containing a single axon and multiple dendrites, which play different roles in electrochemical signaling. Axon and dendrites differ in their molecular composition and precise localization of proteins to the right domains is crucial for neuronal development and neuronal signaling (Bentley and Banker, 2016). Kinesins, dyneins and myosins are molecular motors that facilitate transport of cargoes in cells. Kinesins and dyneins use the microtubule cytoskeleton to facilitate long-distance transport across the cell, where kinesin motors move toward the microtubule plus-ends and dyneins towards the microtubule minus-end (Hirokawa et al., 2010). Kinesin motors typically consist of a motor domain that uses the energy from ATP hydrolysis to walk along the microtubule and a tail domain that interacts with cargo (Hirokawa and Noda, 2008; Kato et al., 2018). So far, 45 different mammalian kinesin genes have been identified and classified into 14 families (Miki et al., 2001; Lawrence et al., 2004). The translocation patterns of different kinesins have been identified using constitutively active motors (Huang and Banker, 2012; Yang et al., 2016) and by using an inducible dimerization system to link a constitutively active motor to an artificial cargo (Lipka et al., 2016). These studies have shown that most kinesins enter both axons and dendrites, but some have an axonal preference.

To ensure proper cargo localization, neurons have developed different cargo sorting pathways. Interestingly, it has been shown that dendritic cargo is restricted to dendrites and vesicles do not enter the axon (Burack et al., 2000; Farías et al., 2012). In contrast, axonal proteins are transported into both axons and dendrites, but cargo trafficking is biased to the axon (Burack et al., 2000; Nakata and Hirokawa, 2003). Tools that manipulate polarized cargo trafficking have provided more insights into the importance of proper cargo sorting for neuronal function. For example, by using nanoparticles and magnetic forces it has been shown that reversing endosomal trafficking affects neurite outgrowth and growth cone motility (Steketee et al., 2011). Furthermore, optogenetic tools that control mitochondrial and endosomal trafficking in neurons have shown that local positioning of recycling endosomes contributes to axon outgrowth (Van Bergeijk et al., 2015). However, these nanobiological and optogenetic tools are technically demanding and require optimization. Therefore, general and easily adaptable tools to study and manipulate polarized cargo trafficking are of interest.

To solve the ‘cargo problem’, for example, addressing which cargo is transported by which kinesin (Terada and Hirokawa, 2000), different approaches have been used. Yeast two-hybrid and immunoprecipitation experiments have yielded potential kinesin-binding partners and identified adaptors that link kinesin motors and cargoes (Hirokawa and Noda, 2008). However, the major limitation of these binding and biochemical assays is the lack of a native cellular environment. For example, the kinetics, thermodynamics, stoichiometry and cofactors, such as lipid membranes, that may affect the motor–cargo interaction are usually not present in these systems. Another approach is to disrupt kinesin motor function in cells by RNAi approaches and examine changes in cargo proteins, thereby providing information about the kinesin–cargo interaction (Hirokawa et al., 2010). The drawback of this approach has been the redundancy in the intracellular transport system. Often one cargo is transported by multiple motors and cells have developed compensatory mechanisms to drive cargo transport, and therefore false-negative results easily arise using these strategies. Recently, a split kinesin method was developed to identify kinesin motors that interact with a fluorescently labeled cargo (Jenkins et al., 2012). Although providing an elegant method to identify kinesin–cargo interaction, the assay is limited by its dependence on single-cell imaging making the experiments time consuming and technically challenging.

Here, we used the principles of the split kinesin method (Jenkins et al., 2012) to develop a broad and highly adaptable toolbox of engineered kinesin motors to study motor–cargo interactions using chemical induction, and we explored different applications to gain insights into the regulation of polarized transport in neurons. Using a systematic imaging platform in fixed neurons, we demonstrated several applications of the toolbox. First, we screened a library of kinesin tail domains for an interaction with one specific cargo. Second, we screened one specific tail domain for an interaction with different cargoes. Finally, we use the assay to further investigate the KIF16B interaction with both dense core vesicles (DCVs) and endosomal vesicles by using different tail domain fragments, point mutations and kinesin chimera constructs. In addition to the investigation of specific kinesin–cargo interactions, our toolbox enabled the manipulation of polarized trafficking of cargoes in living neurons and, by using the motor domains of KIF5C and KIF1A, we were able to successfully steer cargo into axonal and dendritic tips. Our engineered motors provide an efficient and powerful tool for studying kinesin–cargo interactions in living neurons.

## RESULTS

### Engineered motors to identify kinesin–cargo interactions

**Fig. 1.**
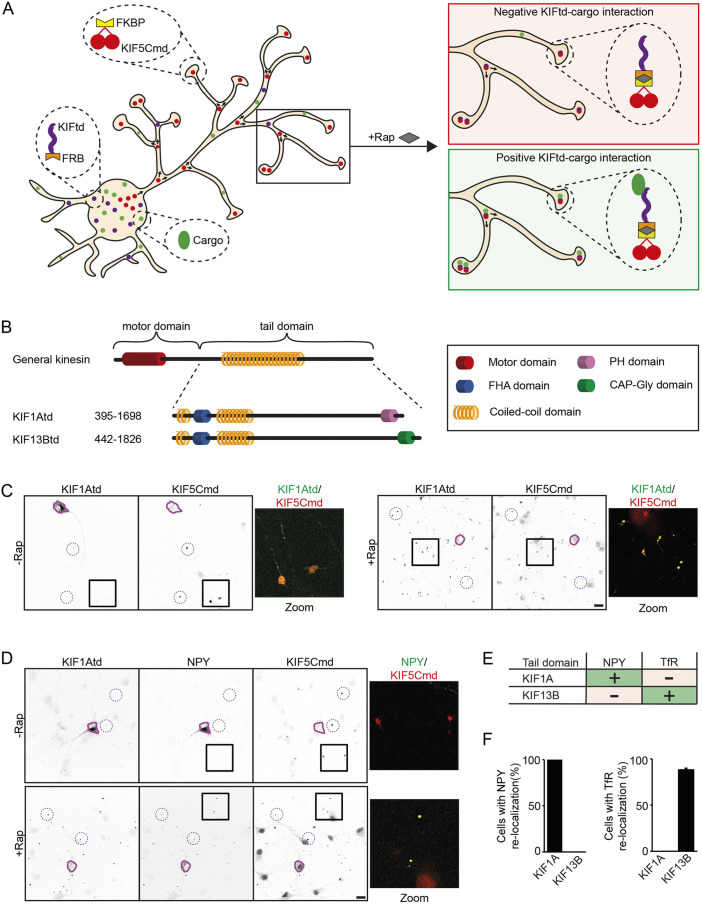
**Kinesin engineering as a tool to identify kinesin–cargo interactions.** (A) Schematic representation of the engineered kinesin assay. Components of the assay include KIF5Cmd fused to an FKBP domain, a kinesin tail domain fused to an FRB domain, and a fluorescently labeled cargo. Without rapalog, the kinesin tail domain and cargo will be diffuse over the cell, whereas the KIF5Cmd will translocate into distal axonal tips. Addition of rapalog induces binding of the FKBP and FRB domains thereby generating a fully functional motor that re-localizes the kinesin tail domain into axonal tips. When there is no interaction between the kinesin tail and the cargo, the cargo will maintain its diffuse localization, whereas in case of an interaction the cargo will also re-localize into distal axonal tips. (B) Overview of the structure of the KIF1A and KIF13B tail domains. (C) Representative images of hippocampal neurons co-expressing FKBP–mRFP–KIF5Cmd and FRB–3myc–KIF1Atd without (left) or with (right) addition of 1 µM rapalog at transfection. The purple line shows the outline of the cell soma and blue dotted circles indicate examples of axonal tips. A merged magnified view of the boxed regions is shown. (D) Representative images of hippocampal neurons co-expressing FKBP–mRFP–KIF5Cmd, FRB–3myc–KIF1Atd and NPY–GFP without (top) or with (bottom) addition of 1 µM rapalog at transfection. The purple line shows the outline of the cell soma and blue dotted circles indicate examples of axonal tips. A merged magnified view of the boxed regions is shown. (E) Overview of the identified interactions between DCVs or endosomal vesicles and KIF1Atd or KIF13Btd in the engineered kinesin assay. (F) Quantification of the percentage of cells in which NPY vesicles or TfR vesicles are re-localized to distal axonal tips in the assay by KIF1Atd or KIF13Btd. Results are mean±s.e.m. (*N*=2 independent experiments, *n*=60 cells). Scale bars: 20 µm.

We based the development of our engineered motor platform on the principle of the split kinesin method (Jenkins et al., 2012). Accordingly, we used the KIF5C motor domain (KIF5Cmd), which in neurons is constitutively active, traffics only into the axon and accumulates in distal axonal tips (Jacobson et al., 2006). Therefore, upon chemical linkage, the kinesin tail domain (KIFtd), which is unable to drive transport on its own, will be translocated into distal axonal tips. Adding a fluorescently labeled cargo to the system allows the identification of interactions between the KIFtd and cargo. In case of an interaction, a marked re-localization of the cargo into the axonal tips will be observed after addition of rapalog (Fig. 1A). We first validated the approach using two previously reported interactions of KIF1A with DCVs (Lo et al., 2011) and KIF13B with transferrin receptor (TfR)-containing vesicles (Jenkins et al., 2012). Therefore, we generated 3myc-tagged KIF1Atd and KIF13Btd constructs (Fig. 1B) fused to an FKBP12–rapamycin-binding (FRB) domain. We tested the system by expressing KIF5Cmd, which was fused to an FKBP domain and mRFP, and either KIF1Atd or KIF13Btd in cultured hippocampal neurons in two different conditions, one without rapalog and one where rapalog was added to the culture medium directly after transfection. Neurons were fixed 1 day after transfection and visualized by fluorescence microscopy. In the absence of rapalog, KIF5Cmd clearly accumulated in distal tips, whereas the KIF1A and KIF13B tails were diffuse over the cell. Upon rapalog treatment the KIF tail domains re-localized into distal axonal tips where they colocalized with KIF5Cmd, verifying that rapalog induced binding between KIF5Cmd and KIFtd, thereby generating a fully functional and non-auto-inhibited kinesin motor (Fig. 1C; Fig. S1A). Next, we verified the interaction of these kinesin tails with GFP-labeled cargo. Cultured hippocampal neurons were transfected with KIF5Cmd, KIFtd, and GFP-tagged Neuropeptide Y (NPY) to label DCVs or GFP-tagged TfR to label endosomal vesicles and treated with or without rapalog. Upon rapalog treatment, we observed a clear re-localization of cargo into distal tips, where it colocalized with KIF5Cmd and KIFtd, and we conclude that rapalog induced transport of NPY-positive DCVs by KIF1Atd and transport of TfR containing endosomal vesicles by KIF13Btd (Fig. 1D; Fig. S1B). In contrast, KIF13Btd was not able to re-localize NPY vesicles and there was no TfR re-localization by KIF1A, thereby providing negative controls (Fig. S1C,D). Overall, these data show that the engineered kinesin motor assay allows to reveal specific interactions between kinesins and their cargo in fixed hippocampal neurons.

### Evaluation of kinesin–cargo interactions in fixed neurons

**Fig. 2.**
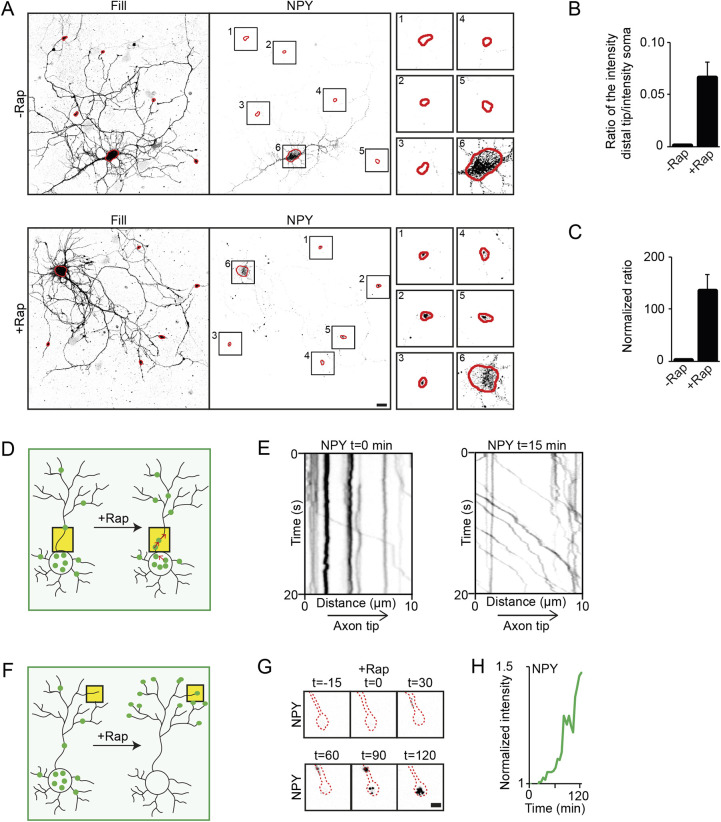
**Quantification of kinesin–cargo binding.** (A) Representative example of the quantification method of the ratio of cargo intensity in distal tips over the soma in neurons co-expressing FKBP–mRFP–KIF5Cmd, FRB–3myc–KIF1Atd and NPY–GFP. Five random distal tips and the soma are selected from the fill (HA–β-galoctidase) for both the conditions without (top) or with (bottom) rapalog (outlined in red). The same regions of interest are then selected in the cargo channel and cargo intensity in these regions is measured. Magnified views of the regions of interest are shown in the small right panels. (B,C) Quantifications of the ratio of cargo intensity in the distal tips to the intensity in the cell soma (B) and the ratio when normalized to the condition without rapalog (C) of the example neuron depicted in A. Results are mean±s.e.m. (*n*=1 cell). (D) Schematic depiction of the readout of the engineered kinesin assay in a neuron. Yellow boxes indicate the localization for live-cell imaging in the axon initial segment. (E) Representative kymographs showing movement of NPY vesicles in the axon initial segment before addition of rapalog (left) and 15 min after (right) addition of 1 µM rapalog in neurons co-expressing FKBP–mRFP–KIF5Cmd, FRB–3myc–KIF1Atd and NPY–GFP. (F) Schematic depiction of the readout of the assay in a neuron. Yellow boxes indicate the localization for live-cell imaging in the growth cone. (G) Stills showing the accumulation of NPY in an axonal tip (dotted red line) over time (in minutes) after addition of rapalog in neurons co-expressing FKBP–mRFP–KIF5Cmd, FRB–3myc–KIF1Atd and NPY–GFP. (H) Graph showing the intensity profile of NPY in the axonal tip (shown in G) after addition of rapalog. Scale bars: 20 µm (A), 2 µm (G).

The original split kinesin method used live-imaging as the main approach to quantify kinesin–cargo interaction. As single-cell live imaging is a time consuming and technically challenging process, we explored different ways to quantify kinesin–cargo interaction in fixed neurons. As shown in Fig. 1, treating the cells with rapalog directly after transfection and fixation after 24 h enabled visualization of KIFtd and cargo re-localization in the cases where there was kinesin–cargo interaction. Therefore, one could simply visualize fixed coverslips and classify them as positive for interaction when colocalization of KIF5Cmd, KIFtd and cargo in distal tips is observed in the rapalog-treated condition, or negative for interaction when no re-localization of the cargo is observed after rapalog treatment. This was undertaken for DCV and TfR transport by KIF1Atd and KIF13Btd (Fig. 1E). However, more quantitative measurements of the efficiency of induced kinesin-cargo binding might be required for some research purposes, and therefore we developed two methods to further quantify kinesin–cargo interactions. In the first method, coverslips are screened for neurons that express all three constructs, KIF5Cmd, KIFtd and GFP–cargo. A neuron is classified as positive for interaction when all three constructs colocalize in distal tips or negative when this is not the case. The percentage of cells in which cargo transport by the KIFtd is observed can then be calculated as is shown for DCV and TfR transport by KIF1Atd and KIF13Btd (Fig. 1F). A second method for quantification was based on the cargo intensity in distal tips and soma. In case of a positive tail–cargo interaction, cargo is moved out of the soma into distal tips, which changes the ratio of cargo in distal tips over cargo in the soma. Here, we use the KIF1A interaction with DCVs as an example to show the quantification. Images of fixed neurons expressing KIF5Cmd, KIF1Atd and NPY–GFP, either treated or not treated with rapalog were taken, and five random axonal tips and the cell soma were selected based on the fill (Fig. 2A). Next, cargo intensity in these regions of interest was measured and the ratio of cargo intensity in the distal tips over cargo intensity in the soma was calculated (Fig. 2B). Normalization to the condition without rapalog treatment, shows that, in the case of kinesin–cargo binding, treatment with rapalog results in a higher ratio of cargo intensity in distal tips to that in the soma (Fig. 2C), thereby providing a measure for kinesin–cargo binding. In contrast, neurons expressing KIF5Cmd, KIF1Atd and TfR–GFP do not show profound changes between the ratio of cargo intensity in distal axonal tips to that in the cell soma (Fig. S2A–C), thereby showing that this method quantitatively identifies positive and negative kinesin–cargo interactions. In conclusion, the assay allows both qualitative and quantitative methods to evaluate kinesin–cargo interactions in fixed neurons.

### Assessment of kinesin–cargo interactions by live-cell imaging

In addition to the evaluation of kinesin–cargo interaction in fixed cells, we were also interested in exploring live-cell imaging approaches. Therefore, we designed two approaches to assess motor–cargo interactions by live-cell imaging and applied these to visualize the interaction between KIF1A and DCVs. In the first approach, which is similar to the approach used in the original split kinesin method, we imaged the axon initial segment. In case of an interaction between kinesin and cargo an increase in vesicle entry into the axon is expected upon addition of rapalog (Fig. 2D). In neurons expressing KIF5Cmd, GFP–NPY and KIF1Atd we observed an increased axonal vesicle entry (Fig. 2E; Movie 1). In the second approach, we imaged an axonal tip over time. In case of association with the KIFtd we expect an accumulation of a labeled vesicle population in the tip (Fig. 2F). When imaging hippocampal neurons expressing KIF5Cmd, GFP–NPY and KIF1Atd we indeed observed accumulation of NPY in the axonal tip ∼90 min after addition of rapalog (Fig. 2G,H; Movie 2). Together, these data show that live-imaging provides an effective way to visualize kinesin–cargo interactions in hippocampal neurons.

### Systematic screening to identify which kinesin binds to a cargo of interest

**Fig. 3.**
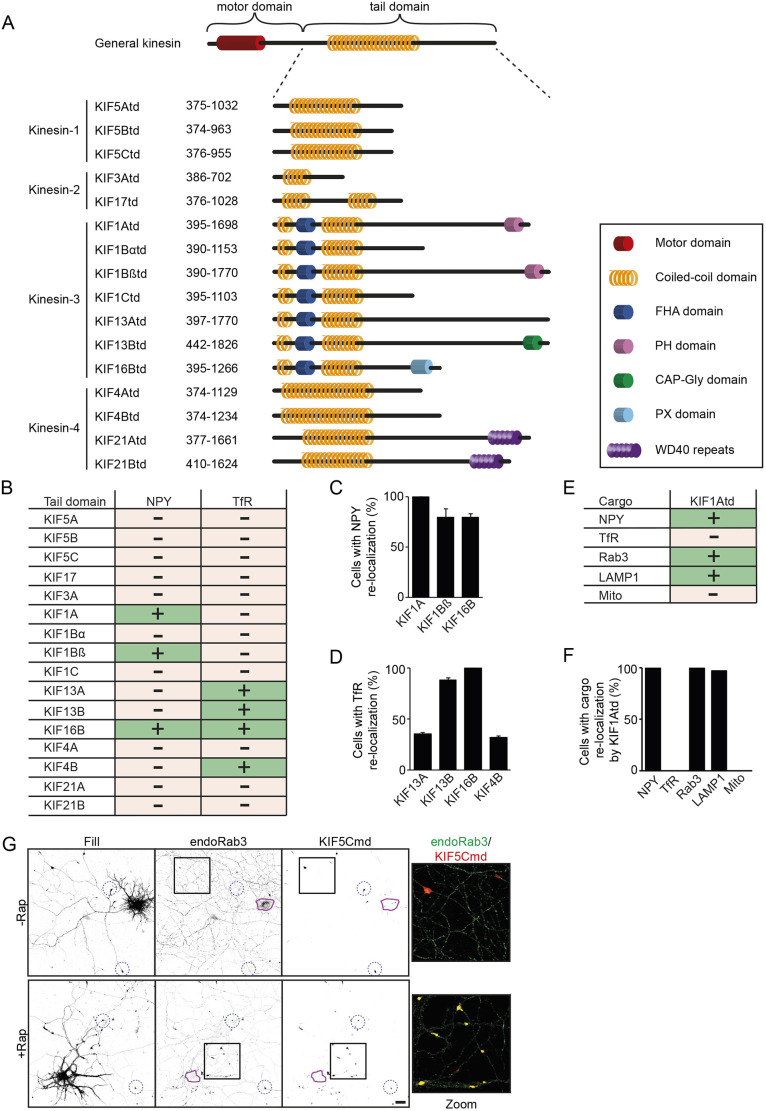
**Screening applications of the engineered kinesin platform.** (A) Overview of the library of kinesin tail domains used to screen for cargo interactions. (B) Overview of the identified kinesins that interact with DCVs and TfR vesicles in the engineered kinesin assay screen. (C,D) Quantification of the percentage of cells in which NPY vesicles (C) or TfR vesicles (D) are re-localized to distal axonal tips in the assay by the identified interacting motors. Results are mean±s.e.m. (*N*=2 independent experiments, *n*=50–60 cells). (E) Overview of the identified cargoes that interact with KIF1Atd in the engineered kinesin assay screen. (F) Quantification of the percentage of cells in which cargo vesicles are re-localized to distal axonal tips by KIF1Atd in the assay. Results are mean±s.e.m. (error=0) (*N*=2 independent experiments, *n*=60 cells). (G) Representative images of a hippocampal neuron co-expressing FKBP–mRFP–KIF5Cmd and FRB–3myc–KIF1Atd and immunostained for Rab3 without (top) or with (bottom) addition of 1 µM rapalog. The purple line shows the outline of the cell soma and blue dotted circles indicate examples of axonal tips. A merged magnified view of the boxed regions is shown. Scale bar: 20 µm.

After developing a variety of analysis methods, we further demonstrate that our toolbox can be used to gather more insights on kinesin–cargo interactions. First, we screened for kinesin–tail interactions with one specific cargo of interest. We generated a library of kinesin tail domain constructs (fused to FRB and tagged with 3myc) in which we included the tail domains of the 16 transporting kinesins from the kinesin-1, kinesin-2, kinesin-3 and kinesin-4 families (Fig. 3A) (Lipka et al., 2016), thereby expanding on the original split kinesin method. Expression of the kinesin tail domains in neurons showed that most tails have a diffuse localization across the neurons, but some, for example, KIF1Atd, KIF1Ctd and KIF16Btd have a clear vesicular-like expression pattern (Fig. S3A). We then tested the rapalog-induced binding for each kinesin tail (examples with KIF5Ctd, KIF17td, KIF16Btd and KIF21Btd are shown in Fig. S3B). Some kinesin tails, for example, KIF5Ctd, were found to be slightly enriched in axonal tips without addition of rapalog. However, these tail domains still showed a large diffuse pool across the cell, and addition of rapalog induced a clear change in the tail domain localization out of the cell soma into distal axonal tips (Fig. S3B). We next wanted to identify all kinesin tails that can interact with DCVs and TfR-containing vesicles and screened our library of tail domains in the assay. Analysis in fixed neurons and quantification of the percentage of cells that showed cargo transport by a KIFtd was the most suitable approach for analyzing such large library screens, and we used this method for analysis of all further experiments. Therefore, fixed neurons were visualized by fluorescence microscopy and classified as positive or negative for interaction. Systematic analysis of all tail domains and both cargoes resulted in an overview of all kinesins that associate with DCVs and TfR vesicles. We identified that DCVs associate with KIF1Atd, KIF1Bβtd and KIF16Btd, whereas TfR vesicles are transported by KIF13Atd, KIF13Btd, KIF16Btd and KIF4Btd (Fig. 3B). For all positive interactions, we quantified the percentage of cells in which kinesin–cargo interaction was observed. For DCVs, transport by KIF1Atd was seen in all cells, whereas ∼80% of the cells showed re-localization by KIF1Bβtd and KIF16Btd, suggesting a slightly weaker interaction between these kinesin tails and DCVs (Fig. 3C). TfR vesicles were very efficiently re-localized by KIF13Btd and KIF16Btd, as, respectively, ∼90% and 100% of cells showed positive interaction. In contrast, for KIF13Atd and KIF4Btd only ∼40% of cells showed efficient cargo re-localization, indicating that these two motors have lower binding affinity for TfR vesicles (Fig. 3D). These results show that the assay provides a robust screening platform to identify kinesin interactions with a specific cargo.

### Systematic screening to identify which kinesin of interest binds to a variety of cargoes

The engineered kinesin motor assay can also be used to screen one kinesin tail domain for an interaction with a variety of organelles, vesicles and other cargoes. Here, we have used KIF1Atd and subjected it to the screening platform using five different cargoes: DCVs, TfR-containing vesicles, synaptic vesicles (SVs, visualized using Rab3A–GFP), lysosomes (visualized using GFP–LAMP1), and mitochondria (visualized using mito-GFP). This screen showed that KIF1Atd interacts with DCVs, SVs and lysosomes, whereas no interaction was observed with TfR vesicles or mitochondria (Fig. 3E,F). In addition to using the assay with overexpressed marker proteins, we were also able to visualize the association of KIF1Atd with endogenous SVs by immunostaining for Rab3A (Fig. 3G). These results show that the engineered kinesin motor assay is a robust tool to identify motor–cargo interactions.

### Identification of the KIF16B PX domain in vesicle association

**Fig. 4.**
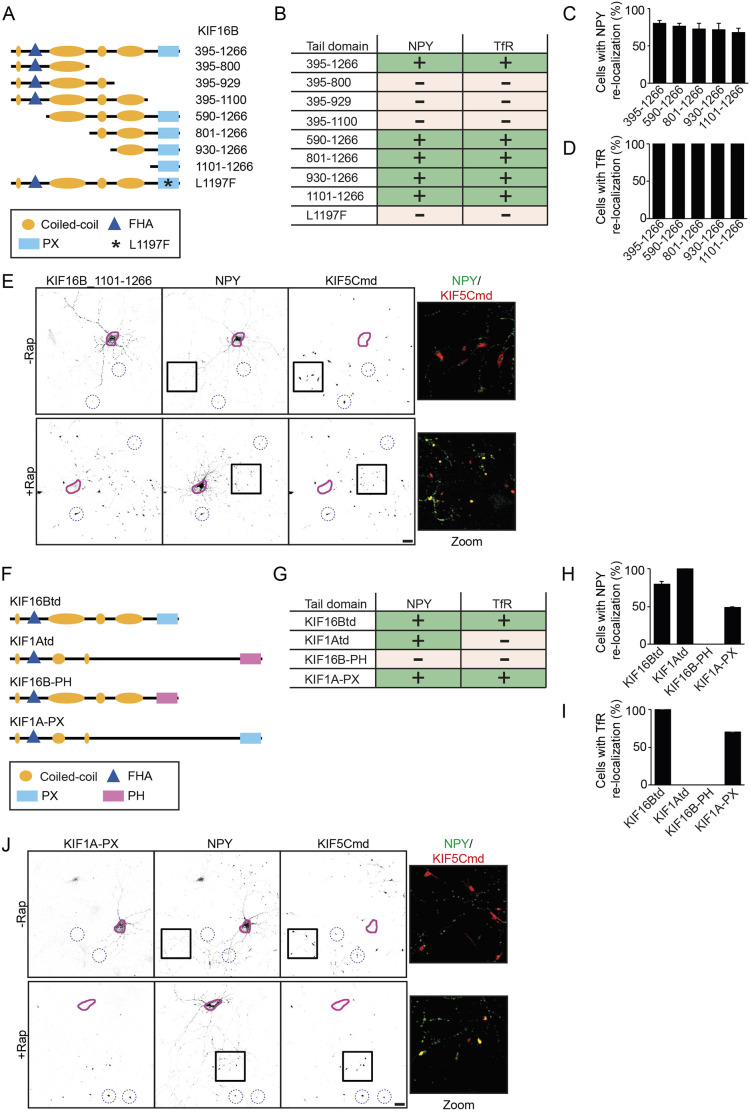
**Validation of the PX domain in the KIF16B-cargo interaction.** (A) Schematic representation of KIF16Btd, truncated KIF16Btd fragments and mutated KIF16Btd. (B) Overview of the different KIF16Btd constructs and their interaction with DCVs and TfR vesicles in the engineered kinesin assay screen. (C,D) Quantification of the percentage of cells in which NPY vesicles (C) or TfR vesicles (D) are re-localized to distal axonal tips in the assay with the identified interacting KIF16Btd fragments. Results are mean±s.e.m. (error=0 in D) (*N*=2 independent experiments, *n*=60 cells). (E) Representative images of hippocampal neurons co-expressing FKBP–mRFP–KIF5Cmd, FRB–3myc–KIF16B_1101-1266 and NPY–GFP without (top) or with (bottom) addition of 1 µM rapalog at transfection. The purple line shows the outline of the cell soma and blue dotted circles indicate examples of axonal tips. A merged magnified view of the boxed regions is shown. (F) Schematic representation of KIF16Btd, KIF1Atd, KIF16B-PH and KIF1A-PX. (G) Overview of KIF16Btd, KIF1Atd and KIF16B–KIF1A chimeras and their interaction with DCVs and TfR vesicles in the engineered kinesin assay screen. (H,I) Quantification of the percentage of cells in which NPY vesicles (H) or TfR vesicles (I) are re-localized to distal axonal tips in the assay by KIF16Btd, KIF1Atd, and KIF16B-KIF1A chimeras. Results are mean±s.e.m. (error=0 in I) (*N*=2 independent experiments, *n*=60 cells). (J) Representative images of hippocampal neurons co-expressing FKBP–mRFP–KIF5Cmd, FRB–3myc–KIF1A-PX and NPY–GFP without (top) or with (bottom) addition of 1 µM rapalog at transfection. The purple line shows the outline of the cell soma and blue dotted circles indicate examples of axonal tips. A merged magnified view of the boxed regions is shown. Scale bars: 20 µm.

We next determined whether our engineered motor platform can be used to develop better mechanistic insight into specific kinesin–cargo interactions. Therefore, we turned our attention to KIF16Btd, which, in our screens, was found to interact both with DCVs and TfR-containing endosomal vesicles. Previously, KIF16B has been implicated in endosomal trafficking and it was found that its PX domain, and specifically L1197, is crucial for the interaction of KIF16B with early endosomes (Farkhondeh et al., 2015). To assess the involvement of the PX domain and L1197 in vesicle association, we generated constructs containing truncated and mutated KIF16Btd fragments linked to FRB and 3myc (Fig. 4A). Expression of these constructs showed that removal of the PX or introducing the L1197F mutation resulted in loss of the KIF16Btd vesicular expression pattern (Fig. S4A). We then screened these KIF16Btd constructs for an interaction with DCVs and TfR endosomal vesicles. Constructs in which the PX domain of KIF16Btd was removed could not associate with either NPY or TfR vesicles, whereas a construct containing only the PX domain was able to interact with both DCVs and endosomal vesicles (Fig. 4B–E; Fig. S1B). Moreover, the L1197F mutation in KIF16Btd abolished vesicle binding, confirming that this specific residue in the PX domain is crucial for vesicle association (Fig. 4B). We further tested whether the PX domain of KIF16B is involved in vesicle binding by generating KIF1A–KIF16B chimeras in which the PH domain of KIF1A and the PX domain of KIF16B are swapped (Fig. 4F). Localization of these constructs showed that swapping the PX domain of KIF16B for the PH domain of KIF1A (KIF16B-PH) resulted in loss of the KIF16B vesicular localization. Interestingly, when the PH domain of KIF1A was replaced by the PX domain (KIF1A-PX) the KIF1Atd adopted the localization of the PX domain (Fig. S4C). As expected, KIF16B-PH was not able to transport NPY or TfR in the assay. However, KIF1A-PX could transport both DCVs and endosomal vesicles (Fig. 4G–J; Fig. S4D). These results suggest that the PX domain of KIF16B is sufficient for the interaction with DCVs and endosomal vesicles, and provides strong evidence of how the engineered motor platform can help to identify specific kinesin tail domain regions critical for cargo binding.

### Different motor domains transport cargo to axon and dendrites

**Fig. 5.**
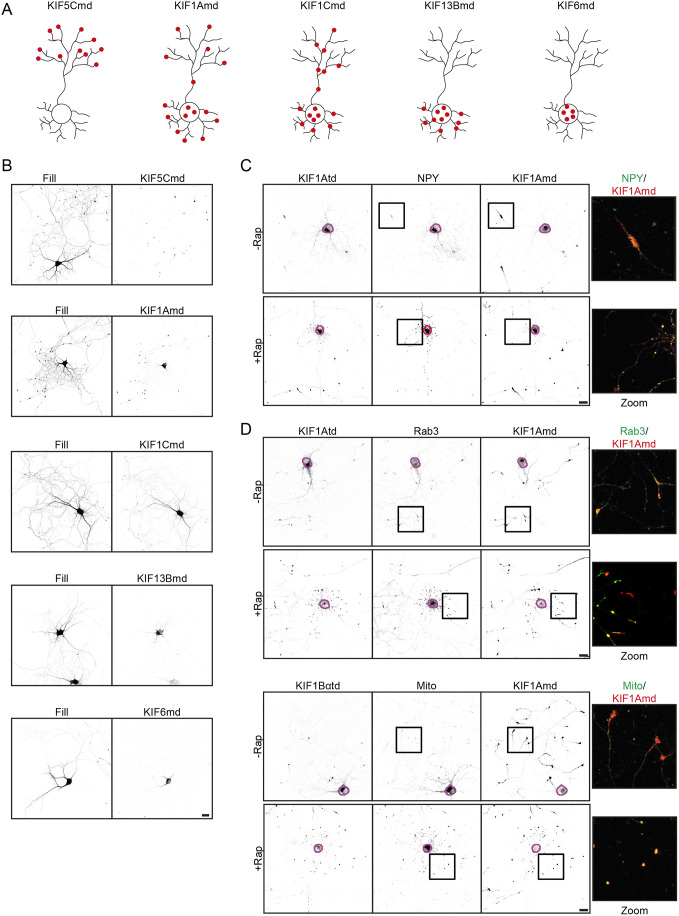
**Different kinesin motor domains drive axonal and dendritic transport.** (A) Schematic representations of the localization of the different motor domains in neurons. (B) Representative images of hippocampal neurons co-expressing GFP (fill) together with FKBP–mRFP–KIF5Cmd, FKBP–mRFP–KIF1Amd, FKBP–mRFP–KIF1Cmd, FKBP–mRFP–KIF13Bmd or FKBP–mRFP–KIF6md. (C) Representative images of hippocampal neurons co-expressing FKBP–mRFP–KIF1Amd, FRB–3myc–KIF1Atd and NPY–GFP without (top) or with (bottom) addition of 1 µM rapalog at transfection. The purple line shows the outline of the cell soma, and a merged magnified view of the boxed regions is shown. (D) Representative images of hippocampal neurons co-expressing FKBP–mRFP–KIF1Amd together with FRB–3myc–KIF1Atd and Rab3–GFP or FRB–3myc–KIF1Bαtd and Mito-GFP without (top) or with (bottom) addition of 1 µM rapalog at transfection. The purple line shows the outline of the cell soma, and a merged magnified view of the boxed regions is shown. Scale bars: 20 µm.

Having shown several applications of our engineered motor platform in the identification of kinesin–cargo interaction, we then wondered whether we could use our approach to artificially induce the targeting of cargoes to other neuronal compartments, such as dendrites or the cell body. To do so, we used the motor domains of KIF1A (KIF1Amd), KIF1C (KIF1Cmd) and KIF13B (KIF13Bmd), which move in both axons and dendrites. Additionally, we used the KIF6 motor domain (KIF6md), which is a non-moving motor (Lipka et al., 2016), to investigate whether we could capture a cargo in the cell soma. We fused the motor domains of KIF1A, KIF1C, KIF13B and KIF6 to mRFP and FKBP and expressed these constructs in hippocampal neurons. We observed that KIF1Amd accumulated in both axonal and dendritic tips, although there was also some motor present along dendrites and axons and in the cell body. KIF1Cmd localized in the cell body and along dendrites and axons, but we did not observe clear accumulation of the motor in distal tips. The KIF13Bmd localized in the somatodendritic region, without accumulation of the motor in distal tips. Finally, KIF6md was localized mainly around the cell body (Fig. 5A,B). Next, we co-expressed KIF1Amd, KIF1Cmd, KIF13Bmd and KIF6md together with KIF1Atd and GFP–NPY, with or without rapalog treatment, and visualized NPY re-localization. Using KIF1Amd, we observed DCV re-localization into both axonal and dendritic tips in all cells (Fig. 5C; Fig. S5B,C). Before addition of rapalog, KIF1Cmd was localized across the cell with no specific accumulation. Interestingly, after rapalog addition and generation of a full motor that interacts with DCVs, KIF1Cmd translocated and accumulated into distal axonal and dendritic tips, suggesting that binding to a tail domain and cargo relieves KIF1Cmd autoinhibition (Fig. S5A–C). When using KIF13Bmd, no clear NPY re-localization was observed, as the motor domain, tail domain and cargo colocalized, but were spread across the cell. We did observe that KIF13Bmd transported into the axon when coupled to the KIF1Atd and interacting with DCVs, whereas the motor domain was mainly somatodendritic in absence of rapalog (Fig. S5D). These observations with KIF1Cmd and KIF13Bmd suggest an additional layer of regulation and/or motor activity when coupled to a kinesin tail and cargo. Using KIF6md in the assay led to a large accumulation of the motor with cargo in the cell soma (Fig. S5E). Together, these results show that inducing the coupling of a given cargo to different kinesin motor domains can be used to control its localization in a specific neuronal compartment.

### Engineered kinesin motors override polarized cargo trafficking in neurons

Using the KIF5Cmd we showed that we were able to re-localize dendritic TfR vesicles into the axon (Fig. S1B). As the KIF1Amd was able to translocate cargo into both axons and dendrites (Fig. 5B), we were wondering whether we could use the engineered kinesin assay to re-localize axonal cargo into dendrites. Therefore, we analyzed the distribution of SVs, which interact with KIF1Atd in our engineered motor assay, and mitochondria, which interact with KIF1Bα (Nangaku et al., 1994). We expressed KIF1Amd together with either GFP–Rab3 and KIF1Atd, or mito-GFP and KIF1Bαtd in hippocampal neurons and subjected these neurons to rapalog treatment. Interestingly, we observed re-localization of both SVs and mitochondria into dendritic tips by KIF1Amd when coupled to KIF1Atd or KIF1Bαtd, respectively (Fig. 5D; Fig. S5F,G). Thus, using engineered kinesin motor cargoes can be re-localized into other neuronal compartments and reverse the polarized cargo sorting in cultured neurons.

## DISCUSSION

### Engineered kinesins allow rapid screening of motor cargo interactions

Cargo-translocation assays have previously been used to study cargo trafficking in neurons (Jenkins et al., 2012; Lipka et al., 2016). However, these assays are limited by their dependence on live-cell imaging, making the experiments technically challenging and time consuming. Here, we developed several methods to visualize and quantify kinesin–cargo interactions in hippocampal neurons by live-cell imaging as well as in fixed cells. We showed that positive cargo interactions result in a clear re-localization of vesicles into axonal tips, which provides an easily interpretable readout in fixed cells and allows systematic detection of kinesin–cargo interactions with high accuracy, without the need for challenging live-imaging experiments, as were mainly used in the original split kinesin method (Jenkins et al., 2012). Furthermore, the fixed approach used in the original method to quantify TfR re-localization from dendrites to the axon depends on a cargo being mainly dendritic before rapalog addition, thereby limiting this analysis method to specific cargo. Thus, our optimized analysis methods expand on the original assay and provide an easily adaptable platform to study a wide variety of kinesin–cargo interaction. In this study, we demonstrated several applications of our optimized platform in the identification of kinesin–cargo interactions. First, we screened a large library of kinesin motors for an interaction with a fluorescently labeled cargo and identified all kinesins that interact with DCVs and TfR-containing endosomal vesicles. Consistent with previous findings, we found interactions of KIF1A with DCVs (Lo et al., 2011) and KIF13B with TfR-containing endosomal vesicles (Jenkins et al., 2012), thereby confirming that the assay successfully identifies known kinesin–cargo associations. Within our laboratory we have also successfully used the platform to identify kinesin interactions with the TrkB receptor (Zahavi et al., 2021). Furthermore, we showed that the engineered kinesin assay can be used to identify interactions of a specific tail domain and a library of various cargoes. In principle, any fluorescently labeled cargo can be subjected to the screen. For instance, we identified DCVs, SVs and lysosomes as cargo for KIF1A, whereas no interaction was found with TfR-containing endosomes or mitochondria. We were also able to visualize the interaction of KIF1Atd with endogenous SVs by immunostaining with anti-Rab3 antibodies. In our screening, we found that KIF16B associates with both DCVs and TfR vesicles, and we further investigated the KIF16B–cargo interaction using different truncated and mutated KIF16B fragments and KIF1A–KIF16B chimeras in the engineered kinesin assay. This revealed that the PX domain, and specifically L1197 within the PX domain, is required for cargo binding, which is in line with previous findings (Farkhondeh et al., 2015). Overall, we show that the engineered kinesin assay provides a robust tool to identify, quantify and study kinesin–cargo interactions.

### Re-localizing polarized cargo into specific neuronal compartments

Precise delivery of proteins into the right neuronal compartment is crucial for neuronal development and function (Bentley and Banker, 2016). Manipulation of the polarized trafficking machinery using nanobiology or optogenetic tools has provided insights into the role of endosomal trafficking in neuronal function (Steketee et al., 2011; Van Bergeijk et al., 2015). However, such techniques require specific technology and optimization, making them difficult to implement. Here, we show that our engineered motor platform enables the selective transport of cargo vesicles upon chemical induction. Our engineered kinesins can re-localize cargo into other neuronal compartments, overriding their endogenous polarized sorting machinery. We show that dendritic TfR-containing vesicles could be re-localized into the axonal compartment using the KIF5C motor domain, whereas typical axonal cargoes such as SVs and mitochondria were re-localized into dendrites by the KIF1A motor domain. Therefore, engineered motors provide an efficient and easy approach to manipulate polarized cargo trafficking. In addition, our toolbox also provides a platform to further study motor function. For example, we observed that the KIF1C motor domain accumulated in distal axonal and dendritic tips only upon chemically induced formation of a full functional motor that interacts with DCVs, suggesting that cargo binding provides an additional layer of regulation of motor activity, potentially by relieving auto-inhibition. Furthermore, the KIF13B motor domain translocated into the axon upon chemically induced linkage to the KIF1A tail domain and interaction with DCVs. Interestingly, full-length KIF13B has been found to mainly interact with dendritically localized cargo (Jenkins et al., 2012). Our data suggest that the KIF13B tail domain contributes to its polarized localization. The engineered kinesin toolbox provides an excellent platform to explore such hypotheses.

### Future applications of the engineered motor proteins

We believe that the toolbox of engineered kinesin motors could be widely used to investigate motor–cargo interactions and we foresee many different applications. The platform could be used to identify an interaction between cargo and any protein of interest. For example, coupling the vesicle-binding regions of the retrograde dynein motor or actin-based myosin motors will allow researchers to identify subunits that bind specific cargoes. Other interesting candidates to be used in the assay are adaptor proteins, which are known to play significant roles in the motor–cargo interaction (Akhmanova and Hammer, 2010; Fu and Holzbaur, 2014). Using adaptor proteins or truncated adaptor fragments in the platform will enable the identification of new adaptors and specific binding regions within an adaptor involved in motor–cargo interaction. Furthermore, the toolbox can be used to identify regulatory mechanisms that are involved in controlling motor–cargo binding, such as Ca^2+^ regulation or phosphorylation by specific kinases. For example, Ca^2+^ levels are proposed to mediate kinesin-1 interaction with mitochondria (Wang and Schwarz, 2009) and phosphorylation of KIF13B by cyclin-dependent kinase 5 mediates the KIF13B association with transient receptor potential vanilloid 1 (TRPV1) (Xing et al., 2012). Performing the assay in low or high Ca^2+^ environments or in the presence of kinase or phosphatase inhibitors could provide new insights into such regulatory mechanisms. A different application might be to use the assay to assess motor strength. It is known that one vesicle can bind multiple motors, which together regulate cargo transport (Gross et al., 2007). By using an additional motor domain, one could induce the generation of a double motor and assess cargo transport. Future studies should investigate motor preference in different cellular environments and stressors to identify regulatory mechanisms in cargo transport by cooperating motors.

### Limitations

The engineered motor platform provides an efficient tool to study kinesin–cargo interactions. The assay depends on re-localization of cargo into distal tips and positive interactions between cargo and kinesin tail domain are efficiently detected. However, a disadvantage of the assay is the possibility to obtain false-negative results. These can arise when the expression level of either the motor or tail domain is low, resulting in only few motors capable of cargo transport. Furthermore, clear re-localization of cargo depends on a tight interaction with the kinesin tail and weaker or transient interactions might not be detected. To decrease the likelihood of obtaining false negatives, we use long-term rapalog treatment. This enables the motor and tail domain to interact with each other directly after protein synthesis and allows enough time for fully functional motors to re-localize cargo into axonal tips, thereby increasing the ability to pick up interactions even at lower expression levels or with weaker interactions. In addition, our quantitative approaches provide a method to pick up interactions even when only few motors are present or in case of weaker binding affinity.

In conclusion, we have developed a highly adaptable toolbox of engineered kinesin motors that allows for the selective transport of specific cargo with chemical induction to study kinesin–cargo interactions. We demonstrate that the assay is robust and has an easily interpretable readout, which enables identification of kinesin–cargo interactions with high specificity and has a wide-range of future applications, thereby providing an excellent toolbox to study kinesin–cargo interactions in living cells.

## MATERIALS AND METHODS

### Animals

Animal experiments were approved by the Dutch Animal Experiments Committee (DEC) and performed according to guidelines of Utrecht University, Dutch law (Wet op de Dierproeven, 1996) and European regulations (Guideline 86/609/EEC). Hippocampal neurons used in this study were obtained from embryonic day 18 (E18) stage embryos of both genders from female pregnant Wistar rats (Janvier) being at least 10 weeks old and were not involved in previous experiments. Rats were housed with a companion in transparent Plexiglas cages with wood-chip bedding and paper tissue. They were kept in a 12-h light–dark cycle with a temperature of 22±1°C and provided with unrestricted access to food and water.

### Primary hippocampal neuron cultures and transfections

Primary hippocampal neuron cultures were prepared from E18 rat brains following protocols described previously (Kapitein et al., 2010a). Neurons were plated in a 12-well plate on coverslips coated with poly-L-lysine (37.5 μg/ml, Sigma) and laminin (1.25 μg/ml, Roche) at a density of 100,000 neurons per well and grown in neurobasal (NB) medium (Gibco) supplemented with 2% B27 (Gibco), 0.5 mM L-glutamine (Gibco), 15.6 µM glutamate (Sigma) and 1% penicillin/streptomycin (Gibco) at 37°C and 5% CO_2_.

Hippocampal neurons were transfected at day *in vitro* 7 (DIV7) using Lipofectamine 2000 (Invitrogen). DNA (1.8 μg/well) was mixed with Lipofectamine 2000 (3.3 μl/well) in 200 μl NB medium and incubated for 30 min. The DNA/lipofectamine mixture was added to the neurons in transfection medium (NB medium supplemented with 0.5 mM glutamine) and incubated for 45 min at 37°C and 5% CO_2_. Neurons were then washed with NB medium and transferred to their original medium at 37°C and 5% CO_2_ until fixation at DIV8. Rapalog (final concentration of 1 µM) was added directly after transfection.

### DNA and shRNA constructs

The following DNA constructs used in this study were as described previously: pβ-actin-HA-β-galactosidase (Hoogenraad et al., 2005), pGW2-TagBFP (Lipka et al., 2016), pGW2-NPY-GFP (Schlager et al., 2010), TfR-GFP (Burack et al., 2000), GFP-LAMP1 (Farías et al., 2017), GFP-Rab3A (van Vlijmen et al., 2008), and Mito-GFP (Hoogenraad et al., 2003). PCR-based strategies were used to clone GW1-KIF5C_1-559-mRFP-(FKBP)_2_ using pBa-Kif5C_1-559-GFP (Addgene plasmid #45059) as a template and ligating it into a GW1-PEX-mRFP-(FKBP)_2_ (Kapitein et al., 2010b) backbone. Similar strategies were used to clone GW1-KIF1A_1-489-mRFP-(FKBP)_2_^­^ (NM_001294149.1), GW1-KIF1C_1-496-mRFP-(FKBP)_2_ (NM_145877.2), GW1-KIF13B_1-444-mRFP-(FKBP)_2_^­^ (NM_015254.4), and GW1-KIF6_1-500-mRFP-(FKBP)_2_ (XM_006244480.1). PCR-based strategies were used to create a GW1-FRB-3myc backbone from the GW1-HA expression vector and GW1-GFP-FRB (Kapitein et al., 2010c). Different KIFtd fragments (Table S1) were cloned into this backbone to generate GW1-FRB-3myc-KIFtd constructs. Truncated KIF16Btd fragments and mutated KIF16Btd were generated using PCR-based strategies with GW1-FRB-3myc-KIF16Btd as template and ligated into the GW1-FRB-3myc backbone. PCR-based strategies with GW1-FRB-3myc-KIF16Btd and GW1-FRB-3myc-KIF1Atd as templates were used to generate KIF1A_395-1559-KIF16B_1101-1266 (KIF1A-PX) and KIF16B_395-1100-KIF1A_1560-1698 (KIF16B-PH), which were cloned into the GW1-FRB-3myc backbone.

### Antibodies and reagents

The following antibodies and dilutions were used in this study for immunofluorescence experiments: mouse anti-Myc (1:200, Bio Connect), chicken anti-β-galactosidase (1:2500, Aveslab), mouse anti-Rab3A (1:200, BD Biosciences), goat anti-chicken-IgY conjugated to Alexa Fluor 405 (1:400, Abcam), goat anti-mouse-IgG conjugated to Alexa Fluor 405 (1:400, Thermo Fisher Scientific), goat anti-mouse-IgG conjugated to Alexa Fluor 488 (1:400, Thermo Fisher Scientific), goat anti-mouse-IgG conjugated to Alexa Fluor 647 (1:400, Thermo Fisher Scientific). For live-imaging analysis NF-CF555 (Farias et al., 2016) was used. A reagent used in this study is rapalog (AP21967, TaKaRa).

### Immunofluorescence staining

Transfected neurons were fixed at DIV8 with 4% formaldehyde and 4% sucrose in phosphate-buffered saline (PBS) at room temperature for 10 min. Cells were then washed three times in PBS-CM (PBS, 1 mM MgCl_2_, 0.1 mM CaCl_2_), permeabilized with 0.2% Triton X-100 for 15 min, and washed one time with PBS-CM, before incubation with 0.2% gelatin for 30 min at 37°C. Next, neurons were incubated with primary antibodies diluted in 0.2% gelatin for 30 min at 37°C, and washed three times in PBS-CM. This was followed by incubation with secondary antibody diluted in 0.2% gelatin for 30 min at 37°C, and washing three times in PBS-CM. Finally, coverslips were mounted in Fluoromount (Invitrogen).

Fixed cells were imaged on: (1) a Nikon Eclipse 80i upright widefield fluorescence microscope, equipped with a Photometrics CoolSNAP HQ2 CCD camera and Nikon NIS Br software, using a Plan Fluor 40× N.A/1.30 oil objective; or (2) a Carl Zeiss LSM 700 confocal laser scanning microscope running ZEN2011 software, using a Plan-Apochromat 40×/1.30 oil DIC objective.

### Image analysis and quantification

#### Classification of kinesin–cargo interaction

To determine which kinesins interact with a cargo, hippocampal neurons transfected with KIF5Cmd, FRB-KIFtd, and GFP-labeled cargo were visualized on a widefield fluorescence microscope. Coverslips were scanned for cells containing all three constructs. If the condition where rapalog was added showed multiple cells with colocalization of KIF5Cmd, KIFtd and cargo in the distal tips, this was classified as kinesin–cargo interaction. If this was not the case, it was classified as no interaction between kinesin and cargo.

#### Quantification of the percentage of cells with cargo transport

Once a kinesin–cargo interaction was identified, neurons expressing KIF5Cmd, FRB-KIFtd and GFP-labeled cargo were visualized on a widefield fluorescence microscope. Coverslips for both the condition without rapalog and the condition with addition of rapalog were scanned for cells containing all three constructs on a first-come-first-serve basis. Cells were classified as transporting if all three constructs colocalized in the distal tips of the cell and, from this, the percentage of cells showing cargo transport was calculated.

#### Quantification of the ratio of cargo intensity in growth cone to that in soma

For quantification of the ratio of cargo intensity in the growth cone to the intensity in the soma, confocal images of neurons transfected with KIF5Cmd, FRB-KIFtd and GFP-tagged cargo were acquired. The fluorescence intensity of the cargo was measured in five growth cones and the cell soma of a hippocampal neuron. This allowed calculation of the ratio of fluorescent intensity in a growth cone to that in the cell soma in the conditions without and with addition of rapalog. The ratio found in the condition with rapalog treatment could be further normalized to the ratio found in the condition without rapalog.

### Live-cell imaging and analysis

Live-cell imaging experiments were performed on an inverted Nikon Eclipse Ti–E confocal microscope equipped with a perfect focus system (Nikon), a CSU–X1–A1 Spinning Disc unit (Yokogawa), a Photometrics Evolve 512 EMCCD camera (Roper Scientific) and a Plan Apo VC 100× N.A.1.40 oil objective. Coverslips were mounted in a Ludin chamber (life imaging services) and maintained in culture medium at 37°C and 5% CO_2_ in a stage incubator (Tokai Hit) during image acquisition.

#### Growth cone imaging

For live-imaging of cargo accumulation in the growth cone, neurons expressing KIF5Cmd, KIF1Atd and GFP–NPY were visualized, and growth cones of different neurons were selected. Images of growth cones were acquired every 5 min for a total of 3 h. After four acquisitions (15 min, *t*=0) rapalog was added to a final concentration of 1 µM. Movies were processed using Fiji (Schindelin et al., 2012) and NPY intensity in the distal tip (determined from KIF5Cmd localization) at different time points was measured.

#### Imaging of axonal vesicle entry

Neurons expressing KIF5Cmd, KIF1Atd and GFP-NPY, were incubated for 30 min with NF-CF555, before imaging. Axons were identified from NF-CF555 staining, and movies in the GFP channel were acquired as stream acquisition with 10 frames/s for 20 s. Movies were taken before addition of rapalog and 15 min after addition of rapalog (1 µM final concentration). Kymographs were generated using the Kymoreslicewide (GitHub: https://github.com/ekatrukha/KymoResliceWide) plugin for Fiji.

## Supplementary Material

Supplementary information

Reviewer comments
