# Supplementary material for: Inducible manipulation of motor–cargo interaction using engineered kinesin motors
Source: J Cell Sci. 2021 Aug 3;134(15):jcs258776. doi: 10.1242/jcs.258776 (PMC8353518; doi:10.1242/jcs.258776)
Supplement: Supplementary information [file joces-134-258776-s1.pdf]

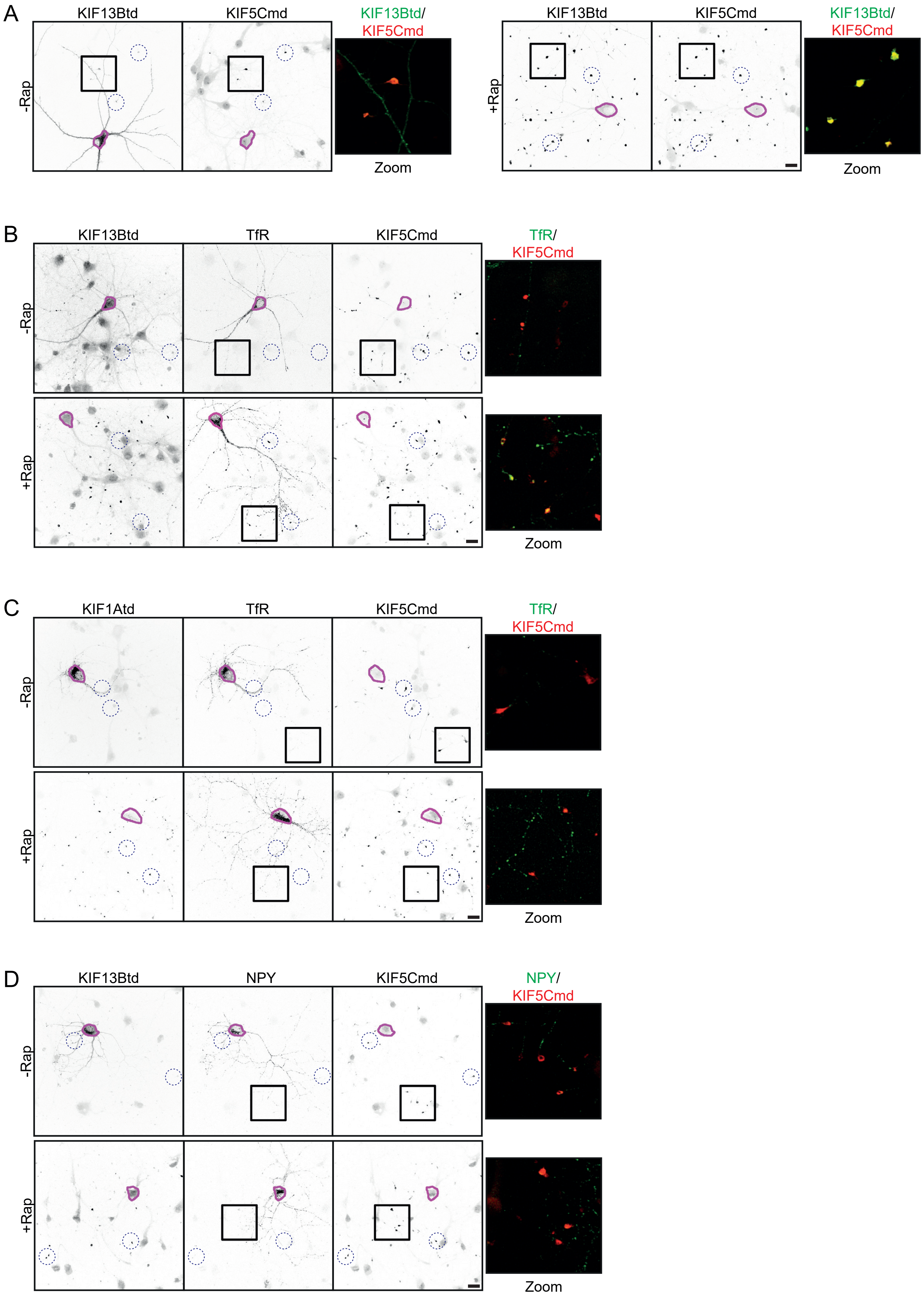

A
KIF13Btd
KIF5Cmd
KIF13Btd/
KIF5Cmd
-Rap
Zoom
KIF13Btd
KIF5Cmd
KIF13Btd/
KIF5Cmd
+Rap
Zoom
B
KIF13Btd
TfR
KIF5Cmd
TfR/
KIF5Cmd
-Rap
+Rap
Zoom
C
KIF1Atd
TfR
KIF5Cmd
TfR/
KIF5Cmd
-Rap
+Rap
Zoom
D
KIF13Btd
NPY
KIF5Cmd
NPY/
KIF5Cmd
-Rap
+Rap
Zoom

Fig. S1. Negative interactions in the engineered kinesin assay

(A) Representative images of hippocampal neurons co-expressing FKBP-mRFP-KIF5Cmd and FRB-3myc-KIF13Btd without (left) or with (right) addition of 1μM rapalog at transfection. The purple line shows the outline of the cell soma and blue dotted circles indicate examples of axonal tips. A merged magnified view of the boxed regions is shown. (B) Representative images of hippocampal neurons co-expressing FKBP-mRFP-KIF5Cmd, FRB-3myc-KIF13Btd and TfR-GFP without (top) or with (bottom) addition of 1μM rapalog at transfection. The purple line shows the outline of the cell soma and blue dotted circles indicate examples of axonal tips. A merged magnified view of the boxed regions is shown. (C, D) Representative images of hippocampal neurons co-expressing FKBP-mRFP-KIF5Cmd, FRB-3myc-KIF1Atd and TfR-GFP (C) or FRB-3myc-KIF13Btd and NPY-GFP (D) without (top) or with (bottom) addition of 1μM rapalog at transfection. The purple line shows the outline of the cell soma and blue dotted circles indicate examples of axonal tips. A merged magnified view of the boxed regions is shown. Scale bars indicate 20 μm.

Journal of Cell Science • Supplementary information

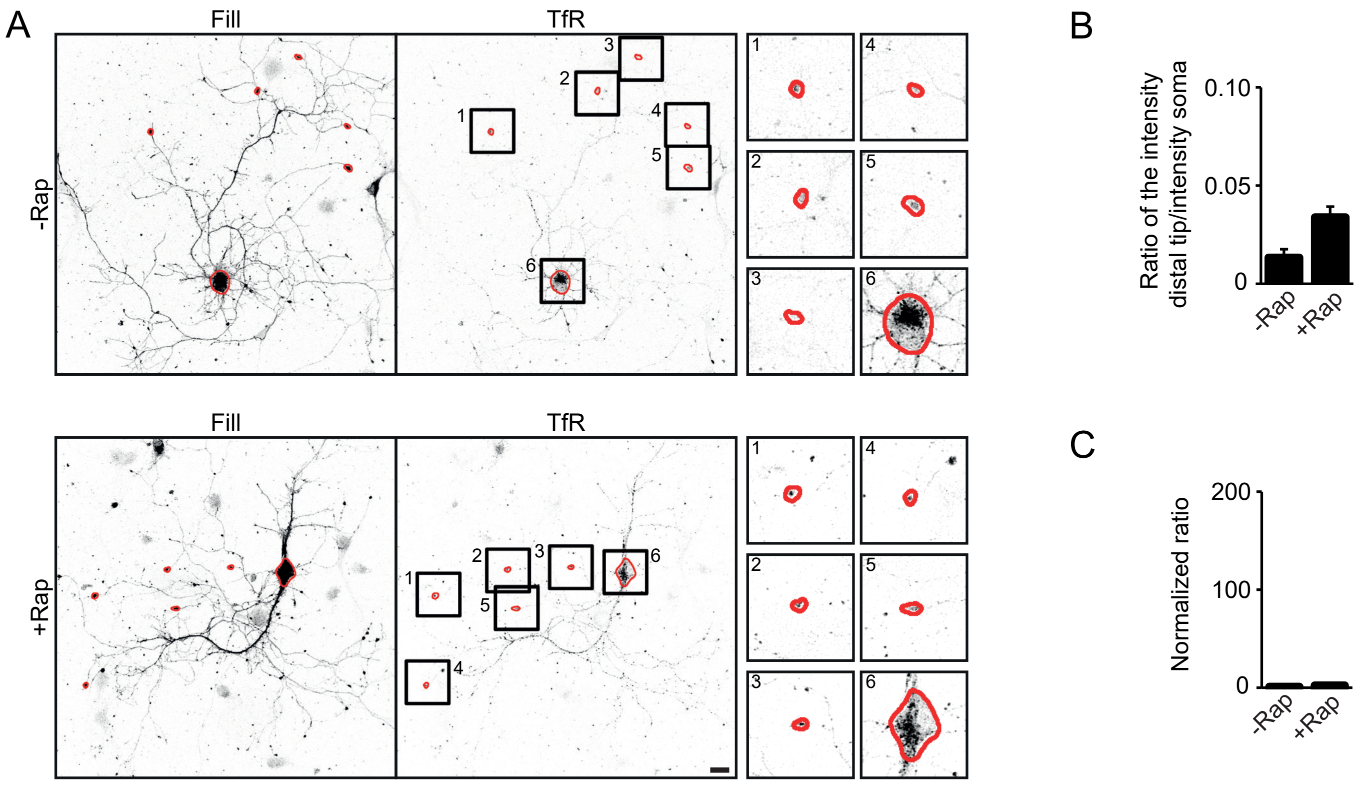

Fig. S2. Quantification of negative kinesin-cargo interaction

(A) Representative example of the quantification method of the ratio of cargo intensity in distal tips over the soma in neurons co-expressing FKBP-mRFP-KIF5Cmd, FRB-3myc-KIF1Atd and TfR-GFP. Distal tips and the soma are selected from the fill (HA-β-galoctidase) for both the conditions without (top) or with (bottom) rapalog (outlined in red). Cargo intensity in these regions of interest is then measured in the cargo channel. Magnified views of the regions of interest are shown in the small right panels. (B, C) Quantifications of the ratio of cargo intensity in the distal tips to the intensity in the cell soma (B) and the ratio when normalized to the condition without rapalog (C) of the example neurons depicted in (A). Results are mean±SEM (n=1 cell). Scale bars indicate 20 µm.

Journal of Cell Science • Supplementary information

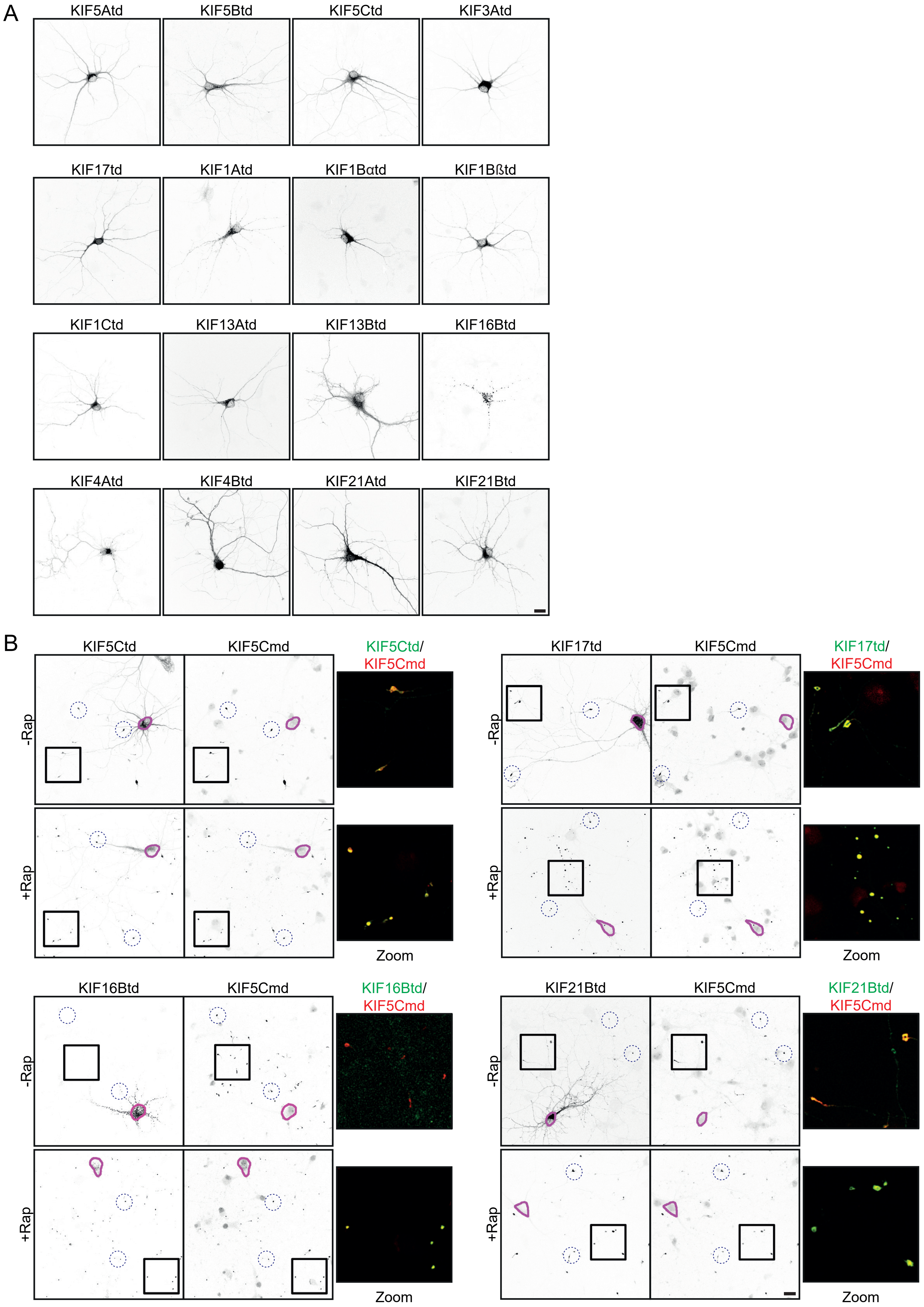

Journal of Cell Science • Supplementary information

Fig. S3. Localization of kinesin tail domains and validation of rapalog-induced binding
(A) Representative images of hippocampal neurons expressing FRB-3myc-KIFtd constructs. (B) Representative images of hippocampal neurons co-expressing FKBP-mRFP-KIF5Cmd and FRB-3myc-KIF5Ctd, FRB-3myc-KIF17td, FRB-3myc-KIF16Btd, or FRB-3myc-KIF21Btd without (top) or with (bottom) addition of 1µM rapalog at transfection. The purple line shows the outline of the cell soma and blue dotted circles indicate examples of axonal tips. A merged magnified view of the boxed regions is shown. Scale bars indicate 20 µm.

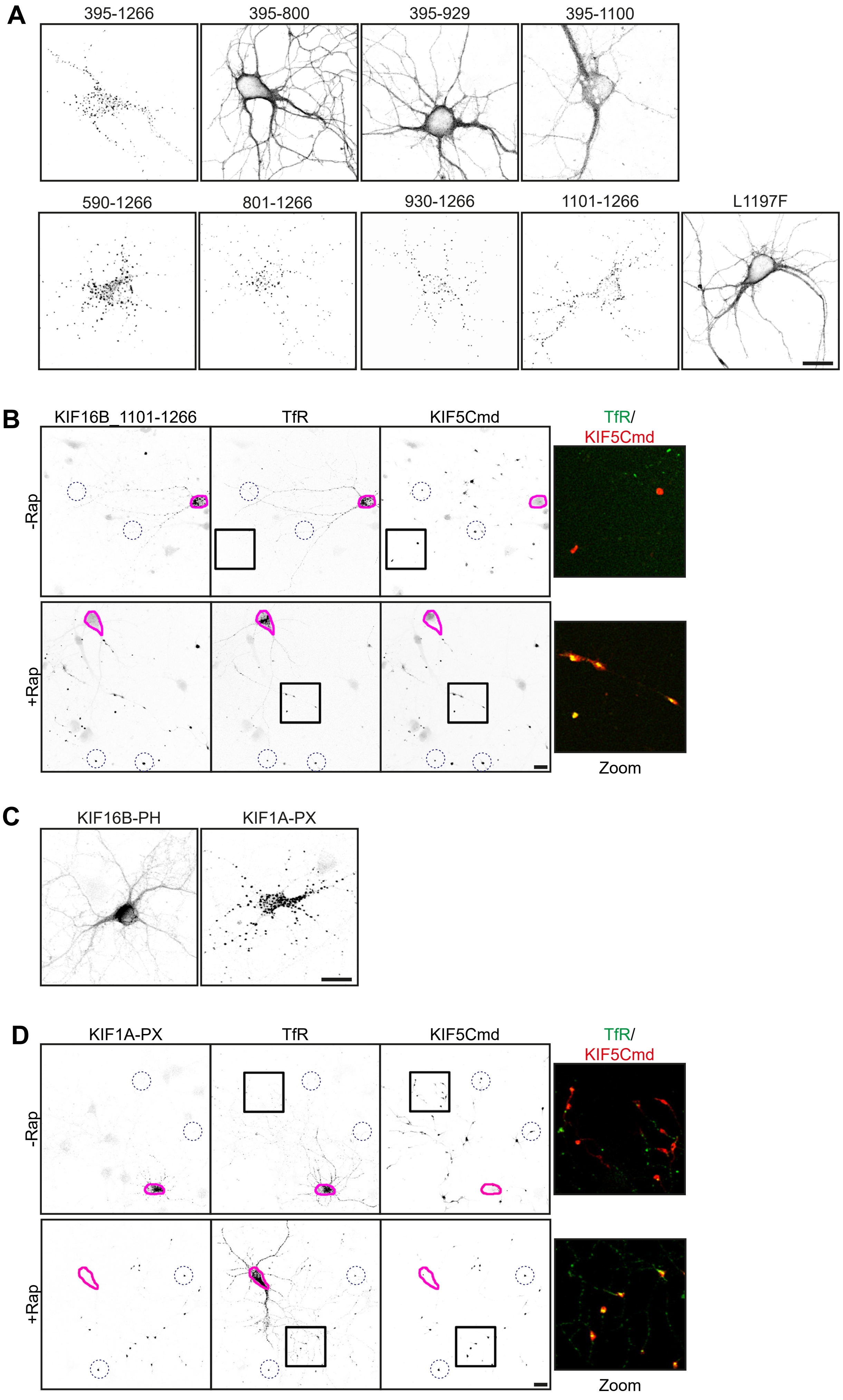

Journal of Cell Science • Supplementary information

Fig. S4. Localization of fragmented, mutated, and chimeric KIF16B constructs (A) Representative images of hippocampal neurons expressing FRB-3myc-KIF16Btd fragments. (B) Representative images of hippocampal neurons co-expressing FKBP-mRFP-KIF5Cmd, FRB-3myc-KIF16B_1101-1266 and TfR-GFP without (top) or with (bottom) addition of 1µM rapalog at transfection. The purple line shows the outline of the cell soma and blue dotted circles indicate examples of axonal tips. A merged magnified view of the boxed regions is shown. (C) Representative images of hippocampal neurons expressing FRB-3myc-KIF16B-PH and FRB-3myc-KIF1A-PX chimera constructs. (D) Representative images of hippocampal neurons co-expressing FKBP-mRFP-KIF5Cmd, FRB-3myc-KIF1A-PX and TfR-GFP without (top) or with (bottom) addition of 1µM rapalog at transfection. The purple line shows the outline of the cell soma and blue dotted circles indicate examples of axonal tips. A merged magnified view of the boxed regions is shown. Scale bars indicate 20 µm.

Journal of Cell Science • Supplementary information

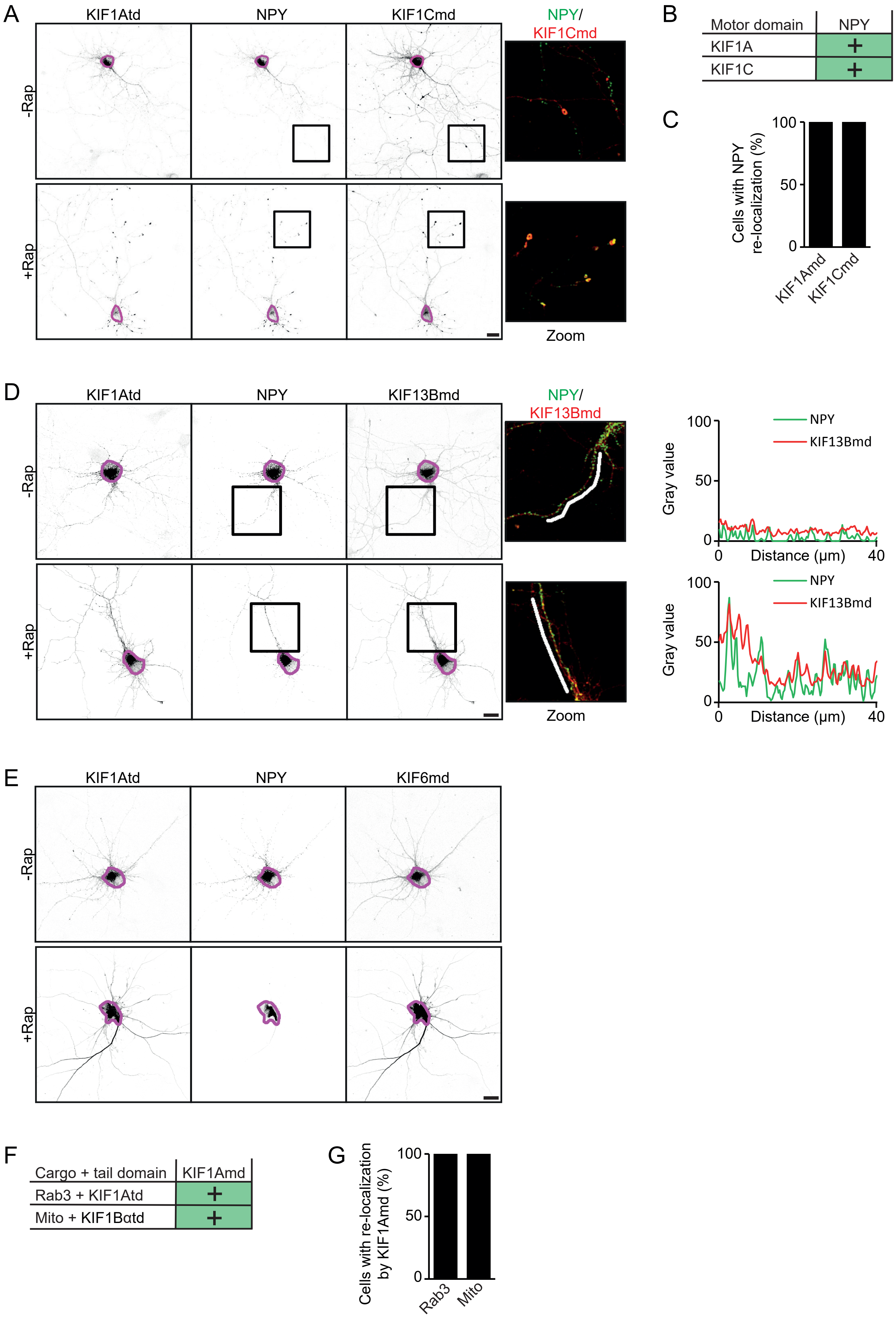

Journal of Cell Science • Supplementary information

Fig. S5. NPY re-localization by KIF13Bmd and KIF6md

(A) Representative images of hippocampal neurons co-expressing FKBP-mRFP-KIF1Cmd, FRB-3myc-KIF1Atd, and NPY-GFP without (top) or with (bottom) addition of 1µM rapalog at transfection. The purple line shows the outline of the cell soma, and a merged magnified view of the boxed regions is shown. (B) Overview of the KIF motor domain constructs and their ability to transport DCVs in the engineered kinesin assay screen. (C) Quantification of the percentage of cells in which NPY vesicles are re-localized to distal axonal tips in the assay by different KIF motor domains. Results are mean±SEM (N=2 independent experiments, n=60 cells). (D) Representative images of hippocampal neurons co-expressing FKBP-mRFP-KIF13Bmd, FRB-3myc-KIF1Atd, and NPY-GFP without (top) or with (bottom) addition of 1µM rapalog at transfection. A merged magnified view of the boxed regions is shown. Graphs show the NPY (green) and KIF13Bmd (red) intensity along the line marked in the zooms. (E) Representative images of hippocampal neurons co-expressing FKBP-mRFP-KIF6md, FRB- 3myc-KIF1Atd, and NPY-GFP without (top) or with (bottom) addition of 1µM rapalog at transfection. (F) Overview of axonal cargo for which dendritic re-localization is observed by KIF1Amd in the engineered kinesin assay screen. (G) Quantification of the percentage of cells in which cargo vesicles are re-localized into dendrites in the assay by KIF1Amd. Results are mean±SEM (N=2 independent experiments, n=60 cells). Scale bars indicate 20 µm.

Journal of Cell Science • Supplementary information

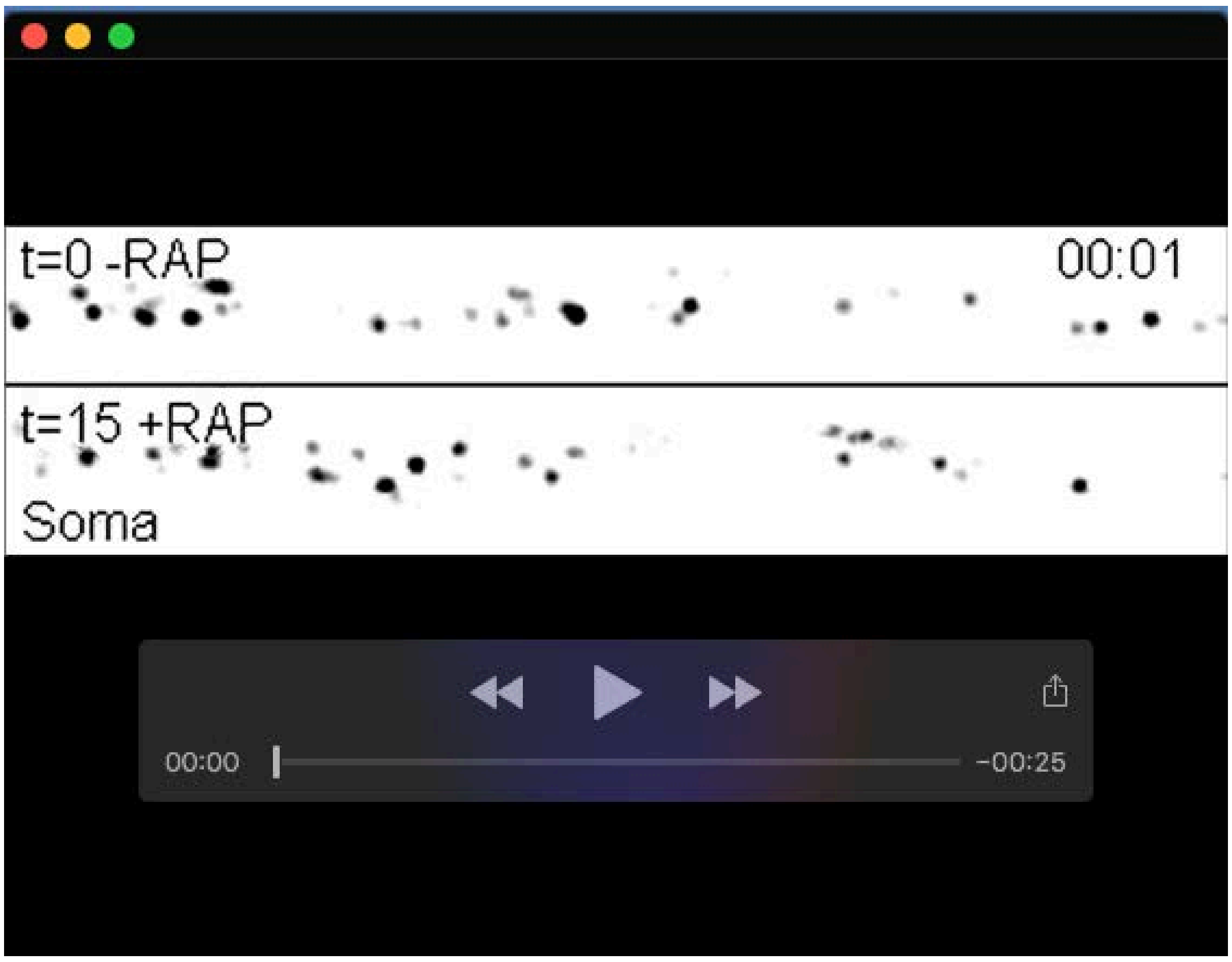

Movie 1. DCV motility in the axon initial segment

Live-imaging of NPY vesicle motility in the axon initial segment in hippocampal neurons co-expressing FKBP-mRFP-KIF5Cmd, FRB-3myc-KIF1Atd and NPY-GFP before or fifteen minutes after addition of rapalog.

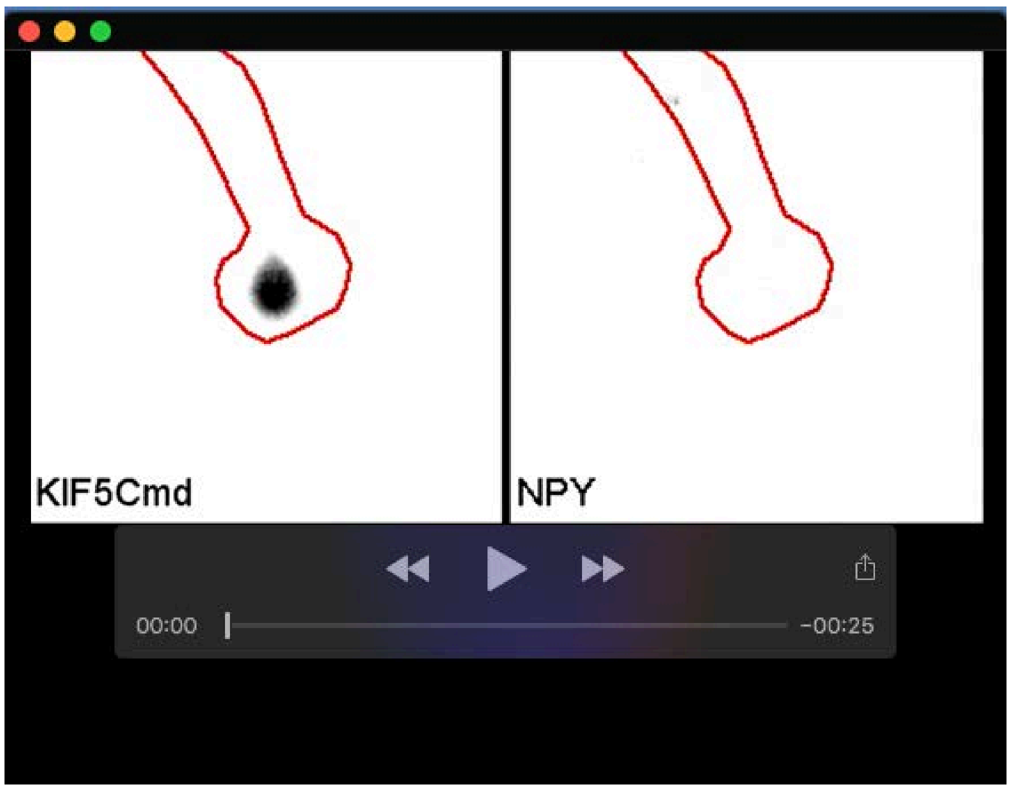

Movie 2. DCV accumulation in axonal tips

Live-imaging of KIF5Cmd-mRFP and NPY-GFP in an distal axonal tip over time after addition of rapalog to hippocampal neurons co-expressing FKBP-mRFP-KIF5Cmd, FRB-3myc-KIF1Atd and NPY-GFP. Red line marks the outline of the distal tip.

Journal of Cell Science • Supplementary information

Table S1. Overview of kinesin tail domains used in this study.

Family	Kinesin	Tail domain	Accession number	Specie
Kinesin-1	KIF5A	375-1032	NM_004984.4	Human
	KIF5B	374-963	NM_004521.3	Human
	KIF5C	376-955	NM_001107730.1	Rat
Kinesin-2	KIF3A	386-702	NM_001300792.2	Human
	KIF17	376-1028	NM_001122819.3	Human
Kinesin-3	KIF1A	395-1698	NM_001294149.1	Mouse
	KIF1Bα	390-1153	NM_001365953.1	Human
	KIF1Bβ	390-1770	NM_015074.3	Human
	KIF1C	395-1103	NM_006612.6	Human
	KIF13A	397-1770	NM_001105566.3	Human
	KIF13B	443-1826	XM_006518620.4	Mouse
	KIF16B	395-1266	NM_001199865.2	Human
Kinesin-4	KIF4A	374-1129	IMAGE: 4538604	Human
	KIF4B	374-1234	NM_001099293.3	Human
	KIF21A	377-1661	NM_017641.4	Human
	KIF21B	410-1624	NM_017596.4	Human
