# Supplementary material for: Inducible manipulation of motor–cargo interaction using engineered kinesin motors
Source: J Cell Sci. 2021 Aug 3;134(15):jcs258776. doi: 10.1242/jcs.258776 (PMC8353518; doi:10.1242/jcs.258776)
Supplement: Reviewer comments [file joces-134-258776_review_history.pdf]

Inducible manipulation of motor-cargo interaction using engineered kinesin motors

Jessica J. A. Hummel and Casper C. Hoogenraad
DOI: 10.1242/jcs.258776

Editor: Giampietro Schiavo

Review timeline

Original submission:	12 April 2021
Editorial decision:	19 May 2021
First revision received:	29 June 2021
Accepted:	1 July 2021

Original submission

First decision letter

MS ID#: JOCES/2021/258776

MS TITLE: Inducible manipulation of motor-cargo interaction using engineered kinesin motors

AUTHORS: Jessica J A Hummel and Casper Hoogenraad
ARTICLE TYPE: Tools and Resources

We have now reached a decision on the above manuscript.

To see the reviewers' reports and a copy of this decision letter, please go to: https://submit-jcs.biologists.org and click on the 'Manuscripts with Decisions' queue in the Author Area. (Corresponding author only has access to reviews.)

As you will see, the reviewers gave favourable reports but raised some critical points that will require amendments to your manuscript. I hope that you will be able to carry these out because I would like to be able to accept your paper.

We are aware that you may be experiencing disruption to the normal running of your lab that makes experimental revisions challenging. If it would be helpful, we encourage you to contact us to discuss your revision in greater detail. Please send us a point-by-point response indicating where you are able to address concerns raised (either experimentally or by changes to the text) and where you will not be able to do so within the normal timeframe of a revision. We will then provide further guidance. Please also note that we are happy to extend revision timeframes as necessary.

Please ensure that you clearly highlight all changes made in the revised manuscript. Please avoid using 'Tracked changes' in Word files as these are lost in PDF conversion.

I should be grateful if you would also provide a point-by-point response detailing how you have dealt with the points raised by the reviewers in the 'Response to Reviewers' box. Please attend to all of the reviewers' comments. If you do not agree with any of their criticisms or suggestions please explain clearly why this is so.

© 2021. Published by The Company of Biologists under the terms of the Creative Commons Attribution License (https://creativecommons.org/licenses/by/4.0/).

Reviewer 1

Advance summary and potential significance to field

see below

Comments for the author

This work by Hummel and Hoogenraad is a highly innovative approach to a problem that has haunted the field for many decades. The power and flexibility to apply the system to different aspects of the axonal/dendritic transport problem is beautifully demonstrated. I strongly support publication, but would recommend to consider a few improvements that would enhance the quality significantly: Firstly, images are far too small and lack symbols (see details below). Secondly, the quantitative analyses proposed should be applied to the studies presented to give the reader a clear impression as to how reliable the tool works (see also comments below). Thirdly, I am asking for some discussion about autoinhibition and the "additional layers of regulation" mentioned by the authors (see details below); these aspects are important limitations that readers need to be aware of and could be dealt with in the last section of the discussion. Andreas Prokop

Detailed comments:
p.4, end of 1st para: "study manipulate" – syntax
p.6, middle: upon rapalog tails localise to tip suggesting that the combined construct fails to auto-inhibit. Please, could the authors comment on this in the text. It does not affect the statements of this paper, but is important to be aware of when planning to use this system.

Fig.1: the graphics are very helpful, but images of originals are far too small. I suggest to show one example with large images in Fig.1 and show further data as large images in supplementary materials. Also, the images seem to look convincing, but it would be important to back qualitative statements up by some quantifications. How reproducible are these results in repeats and in different neurons within an experiment? Are you showing the best examples, or do all neurons look this this? Finally, please indicate in the fluoresent images where cell bodies are as well as axons/axon tips. Should be applied to most figures in this manusript.
p.7: KIF6 is not a kinesin that many readers will be familiar with. It would be helpful if authors could characterise its role in a few words and also provide a rationale as to why a non-moving motor was chosen in this particular experimental context. I would have expected it as a very basic control in the first experiment.

Fig.2. Figures are very small, but here the solutions could be close-ups as image insets; also, please indicate the axon with a symbol. Both these aspects should be improved throughout this manuscript. Like in Fig.1, quantitative information should be provided (e.g. scoring/classifying neurons) to tell the reader how reliable these tools work.
p.7: KIF1C localisation; I guess authors mean KIF1Cmd localisation?
p.7: KIF13B localisation: description in its current form is confusing since the MD restricts to a compartment, but fails to accumulate as I would deduce from the figure.
p.7: make clearer in the text that Fig.2A refers to all motor domain constructs.
p.7: The change of KIF1C from cytoplasmic panneuronal to tip localisation upon tail/cargo linkage suggests that the motordomain alone is inactivated. Please, could the authors briefly conclude here and perhaps offer an explanation?
p.7: KIF13Bmd - Fig.S2 would suggest that more motor domain moves out of the cell body, which I think refers to the statement that the authors make a bit later about axonal localisation. Has this been quantified? Also, the statement that md/td/cargo co-localise is difficult to judge from the far too small images. Ideally one would want to see some correlation analyses.
p.7: "additional layer of regulation" - will this be discussed? If so, please refer to discussion for more detail. If not, please make sure it will be discussed.

Fig.7/8: Please, improve images for Rab3 and mito experiments and provide quantitative statements.
Fig.3A-E: Please, indicate how reliable these findings are, ideally add some quantifications.

© 2021. Published by The Company of Biologists under the terms of the Creative Commons Attribution License (https://creativecommons.org/licenses/by/4.0/).

Fig.8/9: The evaluation section does not describe any procedures that would be out of the ordinary and information would be more appropriate for the methods part. I would rather have expected these kinds of methods to have been routinely applied to experiments shown in Figs. 1 and 2.

Fig.8/9: The evaluation section does not describe any procedures that would be out of the ordinary and information would be more appropriate for the methods part. I would rather have expected these kinds of methods to have been routinely applied to experiments shown in Figs. 1 and 2.
p.10, 1st para: please, stick to consistent nomenclature - most statements are about td constructs but the td suffix is mostly missing. -- the term transport here may be misleading after you presented your live imaging results in a previous section. Re-location/localisation might be clearer.
p.11: KIF16B - please use the td suffix where appropriate
p.11: PH-PX swab - the message here would be potentiated by repeating these experiments with the Rab3 cargo which would be expected to correlate with the presence of PH?

Reviewer 2

Advance summary and potential significance to field

This novel toolbox/system provides an effective conditional switch to study cargo trafficking and cargo binding interactions of motor proteins

Comments for the author

In this manuscript by Hummel and Hoogenraad, the authors developed a toolbox to manipulate polarized cargo trafficking. It consists of an engineered kinesin motor domain with characterized movement trajectories that is fused to an FKBP domain. In combination with an FRB-domain fusion protein, this FKBP-motor can be exploited, with Rapamycin/Rapalogue, as dimerization systems. The novelty of the toolbox is that Hummel and Hoogenraad generated different FRB-fusion proteins encompassing (cargo binding) tail domains of different kinesins. Cell expressing the complete dimerization systems will generate, only in the presence of the chemical inducer Rapamycin/Rapalogue, an effective conditional switch to localize proteins that bind to the FRB-kinesin tail domain.

Hummel and Hoogenraad used this system and engineered kinesin motors to manipulate polarized trafficking of cargos in living neurons. In proof of principle experiments, using the motor domain of KIF5C and KIF1A, the authors show successful relocalized cargo into axonal and dendritic tips. They then screen a library of kinesin tail domains for an interaction with one specific cargo, as well as exploiting the system to use one specific tail domain to screen for interactions with different cargos. In the final set of experiments, they use the assay to investigate the KIF16B interaction with both dense core vesicles (DCVs) and endosomal vesicles to study structural requirements of proteins.

This very nice study underscores the validity and specificity of this toolbox as efficient and powerful tool for studying kinesin-cargo interactions in living neurons.
The one criticism I have is that the cartoons as well as the figures can be improved. The cartoon in the first figure is difficult to follow, and may benefit from clarifying components of this ‘conditional cargo targeting system’. It may benefit from using a second cargo-bding domain NOT interacting with the green cargo…as it is not clear why, in this cartoon, the red box is different from the green. As a reader, I would also like to appreciate the results at higher magnification (Figure 1C, D; Figure 2), and will less ‘disruptions’ by closely drawn circles (Figure 3F). Magnifications should be shown in Figure 4C and Figure 5E, J.

Reviewer 3

Advance summary and potential significance to field

This interesting ms describes a toolbox of extensively re-configurable kinesin constructs that enables identification and study of the cargoes hauled by a particular kinesin, or the kinesins that

© 2021. Published by The Company of Biologists under the terms of the Creative Commons Attribution License (https://creativecommons.org/licenses/by/4.0/).

haul a particular cargo. The system works by splitting kinesins into motor domains fused at their C-termini to FKBP and tails fused at their N-termini to FRB, and connecting them in a rapamycin-dependent manner in vivo. When Kif5c is the motor domain, it transports any interacting cargo to axon tips. Either component can be mutated or switched out to explore the roles of other motor domains and other cargo-recognition motifs in transport of a particular cargo to a particular destination.

The approach appears built on earlier work (Jenkins 2012) that used the same rapalog-dependent split- kinesin approach. the current authors do not use the term 'split kinesin'. The current toolbox may have been developed entirely independently, but so far as I can see, most of the tools presented here were originally engineered, validated and reported, almost a decade ago. The demonstration that Kif5C 559 targets split-kinesin constructs under rapamycin control to axon tips, the contrasting behaviour of Kif1a the surprise finding that Kif13B is dendrite- specific, the use of fixed cells. All in the earlier paper.

The earlier work is referenced at multiple points, but not in a way that acknowledges that it established and validated the toolbox being reported here. What the current report does is revalidate and extend and harness this earlier work. Whereas as written, it claims to have originated the approach (see for example last para of conclusions). I think this is careful and valuable work that does includes entirely novel material (eg KIF16B, semi-automated analysis) but it is appreciably less novel than is claimed. It needs a rewrite to clarify agreements and discrepancies with the earlier work and to explain how the approach has been optimised and extended.

Comments for the author

I definitely encourage revision, the work appears carefully done, it just is claiming a level of originality that it doesn't have.
So far as I can see, most of the tools in the toolbox presented here were originally engineered, validated and reported, in Jenkins et al 2012. Kif5C targeting of split-kinesin constructs under rapamycin control to axon tips, the contrasting behaviour of Kif1a, the surprise finding that Kif13B is dendrite-specific. All in the earlier paper. The same scheme for GFP labelling of cargo vesicles, the same 3myc tag in the tail domain constructs.
The earlier work is referenced at multiple points, but not in a way that acknowledges that it established and validated the toolbox being reported here. What the current report does is extend and expand upon this earlier work. Whereas as written, it claims to have originated the approach (see for example last para of conclusions). It is written as though the split kinesin approach is being validated for the first time.
I have been through the ms and attempted to spot where the claimed points were already established by the earlier work, but the authors themselves will have a much better sense of agreements and discrepancies and I propose they be asked to sort this out. I think this is careful and valuable work that warrants publication but it is appreciably less novel than is claimed. It needs a rewrite to clarify agreements and discrepancies with the earlier work and to explain how the approach has been optimised and extended.

Results
Para 1 KIF5C 559-FKBP fusion ?same as in Jenkins et al KIF1Atd ?constructs / tags same as in Jenkins et al.
KIF13Btd ?constructs / tags same as in Jenkins et al. Hippocampal neurons cultured same? Fixed same?
Imaging same?
(Already acknowledges using the same test of DCV and TfR vesicle transport)
Para 2 Use of KIF6 is additional to Jenkins et al & novel. What is the evidence that KIF6 is a non-moving kinesin?
Fig 4 - library of md and td mutants goes appreciably beyond Jenkins et al. allowing better testing of domain-dependent targeting.
Para 3 - relocalising mitochondria is novel?
Fig 2C - KIF1A and KIF1B cargoes relocalise to dendrites but predominantly still going to axons?
para 4 Live cell imaging

© 2021. Published by The Company of Biologists under the terms of the Creative Commons Attribution License (https://creativecommons.org/licenses/by/4.0/).

para 5 fixed cell imaging
.. big improvements in quantification / analysis workflow / throughput versus the earlier work need highlighting
para 6 Cargo screen - expanded library of KIFs (Fig 4B) versus earlier work
para 7 cargo screen - expanded list of cargoes versus earlier work
para 8 - Kif16B PX domain - completely new insight obtained, as noted in abstract. Line 1 typo inside for insight.

Discussion
Para 2 mentions Jenkins et al but only in passing, to criticise reliance on live cell imaging. in fact Jenkins et al did validate fixed cell imaging.
Discussion of future applications and Limitations is very helpful.
Final para again appears to claim origination of the split kinesin approach.

First revision

Author response to reviewers' comments

Point-to-point response

Reviewer 1
This work by Hummel and Hoogenraad is a highly innovative approach to a problem that has haunted the field for many decades. The power and flexibility to apply the system to different aspects of the axonal/dendritic transport problem is beautifully demonstrated. I strongly support publication, but would recommend to consider a few improvements that would enhance the quality significantly: Firstly, images are far too small and lack symbols (see details below). Secondly, the quantitative analyses proposed should be applied to the studies presented to give the reader a clear impression as to how reliable the tool works (see also comments below). Thirdly, I am asking for some discussion about autoinhibition and the "additional layers of regulation" mentioned by the authors (see details below); these aspects are important limitations that readers need to be aware of and could be dealt with in the last section of the discussion. Andreas Prokop

Detailed comments:
p.4, end of 1st para: "study manipulate" – syntax
We thank the reviewer for his comment. In the revised manuscript we have changed the phrasing in this paragraph.

p.6, middle: upon rapalog tails localise to tip suggesting that the combined construct fails to auto-inhibit. Please, could the authors comment on this in the text. It does not affect the statements of this paper, but is important to be aware of when planning to use this system.
We agree with the reviewer that this should be clarified in the text. We therefore included a sentence commenting on this in the revised manuscript.

Fig.1: the graphics are very helpful, but images of originals are far too small. I suggest to show one example with large images in Fig.1 and show further data as large images in supplementary materials. Also, the images seem to look convincing, but it would be important to back qualitative statements up by some quantifications. How reproducible are these results in repeats and in different neurons within an experiment? Are you showing the best examples, or do all neurons look this this? Finally, please indicate in the fluoresent images where cell bodies are as well as axons/axon tips. Should be applied to most figures in this manusript.

We thank the reviewer for his critical comments regarding our figures. In the revised manuscript we have added larger images for all examples of the engineered motor assay throughout the paper. In order to fit all magnified images, we have moved some of the examples from the main figures to supplementary figures. In the revised manuscript we have also added quantitative data for Figure 1. As mentioned in the figure legends, the chosen images are representative examples. These

© 2021. Published by The Company of Biologists under the terms of the Creative Commons Attribution License (https://creativecommons.org/licenses/by/4.0/).

images represent the majority of neurons observed in a certain condition. The fluorescent image is the merged zoom of the boxed regions depicted and therefore does not contain a cell soma. We believe this might not have been entirely clear, therefore we have made this more apparent in the figures in the revised manuscript.

p.7: KIF6 is not a kinesin that many readers will be familiar with. It would be helpful if authors could characterise its role in a few words and also provide a rationale as to why a non-moving motor was chosen in this particular experimental context. I would have expected it as a very basic control in the first experiment.
We have chosen to include the non-moving motor KIF6, to explore the possibility to capture a cargo in the cell soma. In the revised manuscript we have added the rationale for this choice in the corresponding results section.

Fig.2. Figures are very small, but here the solutions could be close-ups as image insets; also, please indicate the axon with a symbol. Both these aspects should be improved throughout this manuscript. Like in Fig.1, quantitative information should be provided (e.g. scoring/classifying neurons) to tell the reader how reliable these tools work.
In the revised manuscript, we have provided larger images for all examples of the engineered motor assay throughout the figures. In addition, we have added quantifications of cargo re-localization in the corresponding supplemental figure.

p.7: KIF1C localisation; I guess authors mean KIF1Cmd localisation?
The reviewer is correct that we mean KIF1Cmd localization. We have adjusted this in the revised manuscript.

p.7: KIF13B localisation: description in its current form is confusing since the MD restricts to a compartment, but fails to accumulate as I would deduce from the figure.
The reviewer is correct in that the motor domain of KIF13B is restricted to the somatodendritic compartment and it fails to accumulate in distal tips. In the revised manuscript, we have adjusted the phrasing to make this more clear.

p.7: make clearer in the text that Fig.2A refers to all motor domain constructs.
In the revised manuscript we have adjusted the corresponding paragraph to make it clearer that this figure refers to all motor domain constructs.

p.7: The change of KIF1C from cytoplasmic panneuronal to tip localisation upon tail/cargo linkage suggests that the motordomain alone is inactivated. Please, could the authors briefly conclude here and perhaps offer an explanation?

We agree with the reviewer that the change from KIF1Cmd from cytoplasmic panneuronal to tip localization suggests that the motor domain is inactivated. It could be that the motor domain is auto-inhibited and that rapalog-induced binding to a tail domain and cargo relieves the auto-inhibition, resulting in an active motor that moves into distal ends. We have commented on this in the revised manuscript.

p.7: KIF13Bmd - Fig.S2 would suggest that more motor domain moves out of the cell body, which I think refers to the statement that the authors make a bit later about axonal localisation. Has this been quantified? Also, the statement that md/td/cargo co-localise is difficult to judge from the far too small images. Ideally one would want to see some correlation analyses.
We agree with the reviewer that point we make about the axonal localization of KIF13B is not clear. To clarify this point we have added larger images as well as a correlation analysis in the revised manuscript.

p.7: "additional layer of regulation" - will this be discussed? If so, please refer to discussion for more detail. If not, please make sure it will be discussed.
In the revised manuscript we have discussed the additional layers of regulation in the discussion.

Fig.7/8: Please, improve images for Rab3 and mito experiments and provide quantitative statements.

© 2021. Published by The Company of Biologists under the terms of the Creative Commons Attribution License (https://creativecommons.org/licenses/by/4.0/).

We have provided larger images for these experiments in the revised manuscript. In addition, we have added quantifications of cargo re-localization in the corresponding supplemental figure.

Fig.3A-E: Please, indicate how reliable these findings are, ideally add some quantifications.
The live-imaging approaches shown in these figures have been used within our lab to quantify the interaction between KIF1Atd and NPY, LAMP1, Rab3 and TfR as cargos (manuscript in preparation)

Fig.8/9: The evaluation section does not describe any procedures that would be out of the ordinary and information would be more appropriate for the methods part. I would rather have expected these kinds of methods to have been routinely applied to experiments shown in Figs. 1 and 2.

In the revised manuscript we have changed the order of our results section and know explained the different quantification methods directly after Figure 1. Thereby we have early on introduced the quantification method used throughout the paper. In the revised manuscript we have also applied these methods to Figures 1 and Figure 2 (has become Figure 5 in the revised manuscript).

p.10, 1st para: please, stick to consistent nomenclature - most statements are about td constructs but the td suffix is mostly missing. -- the term transport here may be misleading after you presented your live imaging results in a previous section. Re-location/localisation might be clearer.
We thank the reviewer for his critical reading. In the revised manuscript we have added the td suffixes where they were missing. We have also changed the word transport to re-localization.

p.11: KIF16B - please use the td suffix where appropriate
We have corrected this in the revised manuscript.

p.11: PH-PX swab - the message here would be potentiated by repeating these experiments with the Rab3 cargo which would be expected to correlate with the presence of PH?
We agree with the reviewer that it would be interesting to repeat these experiments with Rab3 as cargo. We have therefore expressed KIF16B-PH together with Rab3 in our engineered motor assay. However, we did not observe an interaction with Rab3. These results are in line with previous experiments within our lab, where we show that the KIF1A PH alone is not sufficient for cargo interaction (manuscript in preparation).

Reviewer 2
In this manuscript by Hummel and Hoogenraad, the authors developed a toolbox to manipulate polarized cargo trafficking. It consists of an engineered kinesin motor domain with characterized movement trajectories that is fused to an FKBP domain. In combination with an FRB-domain fusion protein, this FKBP-motor can be exploited, with Rapamycin/Rapalogue, as dimerization systems. The novelty of the toolbox is that Hummel and Hoogenraad generated different FRB-fusion proteins encompassing (cargo binding) tail domains of different kinesins. Cell expressing the complete dimerization systems will generate, only in the presence of the chemical inducer Rapamycin/Rapalogue, an effective conditional switch to localize proteins that bind to the FRB-kinesin tail domain.

Hummel and Hoogenraad used this system and engineered kinesin motors to manipulate polarized trafficking of cargos in living neurons. In proof of principle experiments, using the motor domain of KIF5C and KIF1A, the authors show successful relocalized cargo into axonal and dendritic tips. They then screen a library of kinesin tail domains for an interaction with one specific cargo, as well as exploiting the system to use one specific tail domain to screen for interactions with different cargos. In the final set of experiments, they use the assay to investigate the KIF16B interaction with both dense core vesicles (DCVs) and endosomal vesicles to study structural requirements of proteins.
This very nice study underscores the validity and specificity of this toolbox as efficient and powerful tool for studying kinesin-cargo interactions in living neurons.

The one criticism I have is that the cartoons as well as the figures can be improved. The cartoon in the first figure is difficult to follow, and may benefit from clarifying components of this ‘conditional cargo targeting system’. It may benefit from using a second cargo-bding domain NOT interacting with the green cargo…as it is not clear why, in this cartoon, the red box is different from the green. As a reader, I would also like to appreciate the results at higher magnification

© 2021. Published by The Company of Biologists under the terms of the Creative Commons Attribution License (https://creativecommons.org/licenses/by/4.0/).

(Figure 1C, D; Figure 2), and will less ‘disruptions’ by closely drawn circles (Figure 3F). Magnifications should be shown in Figure 4C and Figure 5E, J.

We thank the reviewer for his critical comments regarding our figures and agree that these can be improved. Therefore, in the revised manuscript we have adjusted the cartoon shown in Figure 1A. Furthermore, throughout the paper we have provided larger images of the examples with the engineered motor assay.

Reviewer 3

Advance summary and potential significance to field...
This interesting ms describes a toolbox of extensively re-configurable kinesin constructs that enables identification and study of the cargoes hauled by a particular kinesin, or the kinesins that haul a particular cargo. The system works by splitting kinesins into motor domains fused at their C-termini to FKBP and tails fused at their N-termini to FRB, and connecting them in a rapamycin-dependent manner in vivo. Then Kif5c is the motor domain, it transports any interacting cargo to axon tips. Either component can be mutated or switched out to explore the roles of other motor domains and other cargo-recognition motifs in transport of a particular cargo to a particular destination.

The approach appears built on earlier work (Jenkins 2012) that used the same rapalog-dependent split-kinesin approach. the current authors do not use the term 'split kinesin'. The current toolbox may have been developed entirely independently, but so far as I can see, most of the tools presented here were originally engineered, validated and reported, almost a decade ago. The demonstration that Kif5C 559 targets split-kinesin constructs under rapamycin control to axon tips, the contrasting behaviour of Kif1a, the surprise finding that Kif13B is dendrite- specific, the use of fixed cells. All in the earlier paper.

The earlier work is referenced at multiple points, but not in a way that acknowledges that it established and validated the toolbox being reported here. What the current report does is revalidate and extend and harness this earlier work. Whereas as written, it claims to have originated the approach (see for example last para of conclusions). I think this is careful and valuable work that does includes entirely novel material
(eg KIF16B, semi-automated analysis) but it is appreciably less novel than is claimed. It needs a rewrite to clarify agreements and discrepancies with the earlier work and to explain how the approach has been optimised and extended.

Reviewer 3 Comments for the author...
I definitely encourage revision, the work appears carefully done, it just is claiming a level of originality hat it doesn't have.

So far as I can see, most of the tools in the toolbox presented here were originally engineered, validated and reported, in Jenkins et al 2012. Kif5C targeting of split-kinesin constructs under rapamycin control to axon tips, the contrasting behaviour of Kif1a, the surprise finding that Kif13B is dendrite-specific. All in the earlier paper. The same scheme for GFP labelling of cargo vesicles, the same 3myc tag in the tail domain constructs.

The earlier work is referenced at multiple points, but not in a way that acknowledges that it established and validated the toolbox being reported here. What the current report does is extend and expand upon this earlier work. Whereas as written, it claims to have originated the approach (see for example last para of conclusions). It is written as though the split kinesin approach is being validated for the first time.

I have been through the ms and attempted to spot where the claimed points were already established by the earlier work, but the authors themselves will have a much better sense of agreements and discrepancies
and I propose they be asked to sort this out. I think this is careful and valuable work that warrants publication, but it is appreciably less novel than is claimed. It needs a rewrite to clarify agreements and discrepancies with the earlier work and to explain how the approach has been optimised and extended.

© 2021. Published by The Company of Biologists under the terms of the Creative Commons Attribution License (https://creativecommons.org/licenses/by/4.0/).

Results
Para 1 KIF5C 559-FKBP fusion ?same as in Jenkins et al KIF1Atd ?constructs / tags same as in Jenkins et al.

KIF13Btd ?constructs / tags same as in Jenkins et al. Hippocampal neurons cultured same? Fixed same? Imaging same?

(Already acknowledges using the same test of DCV and TfR vesicle transport)

Para 2 Use of KIF6 is additional to Jenkins et al & novel. What is the evidence that KIF6 is a non-moving kinesin?

Fig 4 - library of md and td mutants goes appreciably beyond Jenkins et al. allowing better testing of domain-dependent targeting.

Para 3 - relocalising mitochondria is novel?

Fig 2C - KIF1A and KIF1B cargoes relocalise to dendrites but predominantly still going to axons?

para 4 Live cell imaging

para 5 fixed cell imaging

.. big improvements in quantification / analysis workflow / throughput versus the earlier work need highlighting

para 6 Cargo screen - expanded library of KIFs (Fig 4B) versus earlier work

para 7 cargo screen - expanded list of cargoes versus earlier work

para 8 - Kif16B PX domain - completely new insight obtained, as noted in abstract. Line 1 typo inside for insight.

Discussion

Para 2 mentions Jenkins et al but only in passing, to criticise reliance on live cell imaging. in fact Jenkins et al did validate fixed cell imaging.

Discussion of future applications and Limitations is very helpful.

Final para again appears to claim origination of the split kinesin approach.

We thank the reviewer for his critical comments regarding our manuscript. We did base our engineered motor assay on the split kinesin method from Jenkins et al. and we used the same motor domain and myc-tagged tail domain constructs. However, our toolbox expands on the original method by providing several easily adaptable quantification methods, a larger screening library and new applications of engineered motors. In the revised manuscript we have made clearer that our toolbox is based on the split kinesin method and acknowledged earlier work appropriately. We have also compared our toolbox with the previous methods and more clearly addressed similarities and new applications throughout the results and discussion section.

<u>Second decision letter</u>

MS ID#: JOCES/2021/258776

© 2021. Published by The Company of Biologists under the terms of the Creative Commons Attribution License (https://creativecommons.org/licenses/by/4.0/).

MS TITLE: Inducible manipulation of motor-cargo interaction using engineered kinesin motors

AUTHORS: Jessica J A Hummel and Casper Hoogenraad

ARTICLE TYPE: Tools and Resources

I am happy to tell you that your manuscript has been accepted for publication in Journal of Cell Science, pending standard ethics checks.

© 2021. Published by The Company of Biologists under the terms of the Creative Commons Attribution License (https://creativecommons.org/licenses/by/4.0/).
